# Comparative genomics and transcriptomics of lineages I, II, and III strains of *Listeria monocytogenes*

**DOI:** 10.1186/1471-2164-13-144

**Published:** 2012-04-24

**Authors:** Torsten Hain, Rohit Ghai, André Billion, Carsten Tobias Kuenne, Christiane Steinweg, Benjamin Izar, Walid Mohamed, Mobarak Abu Mraheil, Eugen Domann, Silke Schaffrath, Uwe Kärst, Alexander Goesmann, Sebastian Oehm, Alfred Pühler, Rainer Merkl, Sonja Vorwerk, Philippe Glaser, Patricia Garrido, Christophe Rusniok, Carmen Buchrieser, Werner Goebel, Trinad Chakraborty

**Affiliations:** 1 Institute of Medical Microbiology, Justus-Liebig-University, Schubertstrasse 81, Giessen, D-35392, Germany; 2 Helmholtz-Zentrum für Infektionsforschung GmbH, Inhoffenstraße 7, Braunschweig, 38124, Germany; 3 Center for Biotechnology, University of Bielefeld, Bielefeld, D-33594, Germany; 4 Institut für Biophysik und physikalische Biochemie, Universität Regensburg, Universitätstrasse 31, Regensburg, D-93053, Germany; 5 Febit Biomed GmbH, Im Neuenheimer Feld 519, Heidelberg, D-69120, Germany; 6 Institut Pasteur, Laboratoire Evolution et Génomique Bactériennes and CNRS URA 2171, Paris, 75724, France; 7 Institut Pasteur, Biologie des Bactéries Intracellulaires and CNRS URA 2171, Paris, 75724, France; 8 Max von Pettenkofer-Institut for Hygiene and Medical Microbiology, Ludwig Maximilians-University München, Pettenkoferstrasse 9a, Munich, D-80336, Germany

**Keywords:** *Listeria monocytogenes*, Lineage, Comparative genomics, Gene decay, Comparative transcriptomics, Flagella, Prophage, Monocin, Isogenic deletion mutants, Murine infection

## Abstract

**Background:**

*Listeria monocytogenes* is a food-borne pathogen that causes infections with a high-mortality rate and has served as an invaluable model for intracellular parasitism. Here, we report complete genome sequences for two *L. monocytogenes* strains belonging to serotype 4a (L99) and 4b (CLIP80459), and transcriptomes of representative strains from lineages I, II, and III, thereby permitting in-depth comparison of genome- and transcriptome -based data from three lineages of *L. monocytogenes*. Lineage III, represented by the 4a L99 genome is known to contain strains less virulent for humans.

**Results:**

The genome analysis of the weakly pathogenic L99 serotype 4a provides extensive evidence of virulence gene decay, including loss of several important surface proteins. The 4b CLIP80459 genome, unlike the previously sequenced 4b F2365 genome harbours an intact *inlB* invasion gene. These lineage I strains are characterized by the lack of prophage genes, as they share only a single prophage locus with other *L. monocytogenes* genomes 1/2a EGD-e and 4a L99. Comparative transcriptome analysis during intracellular growth uncovered adaptive expression level differences in lineages I, II and III of *Listeria*, notable amongst which was a strong intracellular induction of flagellar genes in strain 4a L99 compared to the other lineages. Furthermore, extensive differences between strains are manifest at levels of metabolic flux control and phosphorylated sugar uptake. Intriguingly, prophage gene expression was found to be a hallmark of intracellular gene expression. Deletion mutants in the single shared prophage locus of lineage II strain EGD-e 1/2a, the *lma* operon, revealed severe attenuation of virulence in a murine infection model.

**Conclusion:**

Comparative genomics and transcriptome analysis of *L. monocytogenes* strains from three lineages implicate prophage genes in intracellular adaptation and indicate that gene loss and decay may have led to the emergence of attenuated lineages.

## Background

*Listeria monocytogenes* is a Gram-positive, motile, non-sporulating, rod shaped bacterium. It is the causative agent of listeriosis, a food-borne disease, which afflicts both humans and animals. There are only eight species in the entire genus, *L. monocytogenesL. marthiiL. innocuaL. seeligeriL. welshimeriL. ivanoviiL. grayi* and *L. rocourtiae*. *L. monocytogenes* and *L. ivanovii* are the pathogenic species while the others are apathogenic [1,2]. In the genus *Listeria*, non-pathogenic species have been hypothesized to have evolved through genome reduction from pathogenic progenitor strains [3]. *L. monocytogenes* is able to invade and replicate in both phagocytic and non-phagocytic cells. The infectious life cycle has been elucidated in detail, and several virulence factors, essential for each stage of infection have been identified [4,5]. Pathogenic listeriae encode several virulence factors that are localized in a virulence gene cluster (*vgc*) or *steria*pathogenicity island-1 (LIPI-1) in the genome. However, a number of genes required for virulence are not localized in this cluster, including the two internalins *inlA* and *inlB*. These encode proteins that are expressed on the surface of the bacterium and facilitate the entry of the bacterium into the eukaryotic cell and their incorporation into a membrane-bound vacuole [6,7]. Further pathogenicity islands present in the genus *Listeria* code for multiple internalins and additional hemolysin genes in species *L. ivanovii* (LIPI-2) [8] and a subset of strains of lineage I (LIPI-3) [9].

Within the four lineages of *L. monocytogenes*, strains are generally classified by serotyping or MLST [10,11], of which 1/2a, 1/2b and 4b are most commonly associated with human listerial infections [2,12]. The first outbreak of *L. monocytogenes* was described for the strain EGD-e, a serotype 1/2a strain of lineage II, following an epidemic in rabbits and guinea pigs in 1926 by E.G.D. Murray [13]. This strain has become a model *Listeria* strain, and was the first listerial strain to be completely sequenced, along with the non-pathogenic *Listeria innocua* 6a CLIP11262 [14]. Subsequently, the first genome of a 4b serotype strain (F2365) of lineage I was completely sequenced [14,15]. It was isolated from Jalisco cheese during a listeriosis outbreak in California in 1985 and mainly associated with pregnancy-related cases. However, it has been recently shown that this strain contains nonsense and frameshift mutations in several genes. Owing to a frameshift in *inlB*, F2365 is severely compromised in Caco-2 invasion assays [16].

Here we report thus the genome sequence of a clinical isolate of the 4b serotype of lineage I, the *L. monocytogenes* 4b strain CLIP80459 that was isolated in a clinical outbreak of listeriosis in France affecting 42 persons [17]. We also present the complete genome sequencing of *L. monocytogenes* strain 4a L99 of lineage III. L99 was originally isolated from food by Kampelmacher in 1950s in the Netherlands. This strain is attenuated in its virulence properties and exhibits a restricted ability to grow within the liver and spleen of infected mice [18]. The availability of the complete genome of *L. monocytogenes* EGD-e serotype 1/2a has permitted analysis of the intracellular gene expression profile of this strain [19-21].

The genome sequences of strains 4a L99 and 4b CLIP80459 presented in this work provide a unique opportunity to delineate specific adaptations of these lineage representives both at the genomic and at the transcriptional level.

## Results

### General features of complete genomes of three lineages of *L. Monocytogenes*

The overall features of the completely sequenced circular genome*s* of *L. monocytogenes* 4a L99*, L. monocytogenes* 4b CLIP80459, *L. monocytogenes* 1/2a EGD-e, *L. monocytogenes* 4b F2365 and *L. innocua* 6a CLIP11262 are given in Table 1. Computational multi-virulence-locus sequence typing (MVLST) [22] analysis showed that strain 4b CLIP80459 belongs to epidemic clone ECII and strain 4b F2365 to epidemic clone ECI as previously reported by Nelson and colleagues [15], respectively. The *L. monocytogenes* genomes are remarkably syntenic: genome size, G + C content, percentage coding and average length of protein-coding genes are similar among all four strains (which was previously reported for other listerial genomes) [14,15]. All four *L. monocytogenes* genomes harbour 67 tRNA genes and contain six complete copies of rRNA operons (16 S-23 S-5 S), of which two are located on the right and four on the left replichore. The chromosomes of 4a L99 and 4b CLIP80459 are devoid of mobile genetic elements and harbour no plasmid*.*

**Table 1:** General features of *L. monocytogenes* 1/2a EGD-e, *L. monocytogenes* 4a L99, *L. monocytogenes* 4b CLIP80459, *L. monocytogenes* 4b F2365 and *L. innocua* 6a CLIP11262

	***L. monocytogenes*4a L99**	***L. monocytogenes*4b CLIP80459**	***L. monocytogenes*4b F2365**	***L. monocytogenes*1/2a EGD-e**	***L. innocua*6a CLIP11262**
**Size of chromosome [bp]**	2979198	2912690	2905187	2944528	3011208
**G + C content [%]**	38.2	38.1	38.0	38.0	37.4
**G + C content of protein-coding genes [%]**	38.7	38.5	38.5	38.4	37.8
**Protein-coding genes (pseudogenes)**	2925 (1)	2790 (24)	2821 (26)	2855 (9)	2981 (13)
**Average length of protein-coding genes [aa]**	301	311	303	306	300
**Number of rRNA operons (16 S-23 S-5 S)**	6	6	6	6	6
**Number of tRNA genes**	67	67	67	67	66
**Percentage coding**	88.9	89.4	88.4	89.2	89.2
**Number of prophages (genes)**	4 (191)	1 (16)	1 (16)	2 (79)	6 (322)
**Plasmid**	0	0	0	0	1
Number of strain-specific genes*	111	49	105	120	89
Number of orthologous genes*	2623	2725	2699	2656	2570
**Number of transposons**	0	0	0	1	0
*Prophage genes excepted.					

Core and specific genes were analyzed using orthologous pairs excluding prophage genes as described previously [3].

We observed four different prophage regions in the genome of the 4a L99 and only one in the 4b CLIP80459 strain (see prophage region II). *L. monocytogenes* 4a L99 prophage I is located at position 71438 bp (*lmo4a_0064lmo4a_0115*), prophage II at (*lmo4a_0148lmo4a 0153,* prophage-remnant: *lmaDC*; 4b ClIP80459 Lm4b_00117b-Lm4b00134 or monocin region), prophage III at 1224779 bp (*lmo4a_1221lmo4a*_*1293*) and prophage IV at 2668913 bp (*lmo4a_2599lmo4a_2658*). Two prophage regions, I and III, are located adjacent to tRNAs*.* Prophage region I is flanked by *tRNA*^*Lys*^ and prophage region III is inserted within the region between the gene for *tRNA*^*Arg*^ and *ydeI* compared to *L. monocytogenes* 1/2a EGD-e. At this very chromosomal location in *L. welshimeri* 6b SLCC5334 there is an insertion of a prophage [3,23,24], while *L. ivanovii* harbours the species-specific *Listeria* pathogenicity island 2 (LIPI-2), which contains a sphingomyelinase C (SmcL) and also a cluster of internalin genes [8]. These findings confirm previous observations [3] indicating that tRNAs represent genetic “anchoring elements” for the uptake of listerial prophage DNA by transduction processes and thus contributing to evolutionary genome diversity of listeriae. Pseudogenes were detected for both 4b F2365 (24 pseudogenes) and 4b CLIP80459 (26 pseudogenes) genomes respectively, which is a higher number compared to that seen in *L. monocytogenes* 1/2a EGD-e (9 pseudogenes), *L. monocytogenes* 4a L99 (one pseudogene) and *L. innocua* (13 pseudogenes).

When comparing the two *L. monocytogenes* 4b genomes (CLIP80459 and F2365) 115 genes are specific for strain 4b CLIP80459 with respect to strain 4b F2365. The dominant functions encoded by these genes are related to sugar metabolism as they comprise five PTS systems and five sugar permeases or sugar transporters. Furthermore, four transcriptional regulators and four surface anchored proteins are specific to 4b CLIP80459 indicating differences in regulation, sugar metabolism and surface characteristics between the two strains. Of the 146 genes found to be specific for strain 4b F2365, the majority were of unknown function, apart from a PTS system and a specific surface protein. Most interestingly, *inlB* although it is reported to be important for virulence of *L. monocytogenes* has a frameshift mutation in this strain [15].

**Figure 1:**
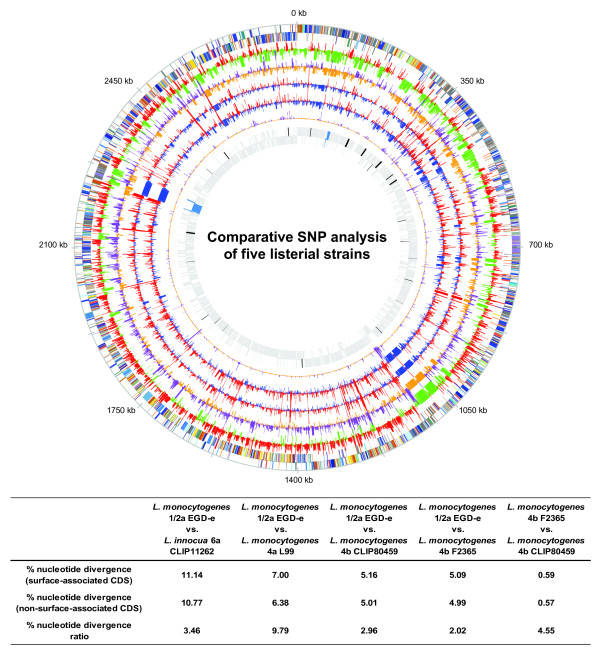
**Comparative SNP analysis of five listerial strains** From outside to inside: genome of *L. monocytogenes* 1/2a EGD-e colored according to COG categories (two strands shown separately). Number of SNPs normalized by gene length in the comparison of 1/2a EGD-e and *L. innocua* 6a CLIP11262, 1/2a EGD-e and 4a L99, 1/2a EGD-e and 4b CLIP80459, 1/2a EGD-e and 4b F2365, and the two 4b strains (4b F2365 and 4b CLIP80459). The innermost circle shows the location of phage genes (blue) and virulence genes (black) in the 1/2a EGD-e genome. Line graphs indicate the number of SNPs/gene length reflecting loci in the genome having a disproportionate number of SNPs. However, if a gene is specific to a certain genome, this will also be shown as a peak indicating a region of divergence within the two genomes under comparison. This analysis was performed using the MUMmer package [25] and SNPs were mapped to coding regions using PERL scripts. Data were visualized by GenomeViz [26]. For each pairwise comparison of strains, percentage of SNPs per gene length of surface- and non-surface-associated genes, as well as the ratio of these values is given in the table. The latter was named “nucleotide divergence ratio” and denotes the relative amount of difference between those two classes of genes, in order to identify more (positive value) or less (negative value) abundant mutation in surface-associated than in non-surface-associated genes.

When comparing the genomes of different lineages at the nucleotide sequence level a number of genomic differences were revealed (Figure 1). Surface proteins showed the highest number of single nucleotide polymorphisms (SNPs). Even in the comparison of the two closely related 4b genomes, two LPXTG-motif containing proteins were identified as encoding a large number of SNPs. One of these, *lm4b_01142* shares substantial similarity to internalins. Comparison of the 4a L99 and the 1/2a EGD-e genomes reflected larger evolutionary divergence, but once again involved surface proteins, such as the LPXTG-motif containing protein *lmo1799*, internalin *lmo0409* (*inlF*)*,* autolysin *lmo1215*, as well as proteins involved in surface antigen biosynthesis like *lmo2552* (*murZ*) and *lmo2549* (*gtcA*). Further analysis identified genes that are most divergent in the three lineages and classification of the most divergent orthologous gene groups was performed (Additional file 1: Table S 1). Thus, distribution of SNPs in *Listeria* suggests considerable evolutionary adaptation among surface-associated genes.

### Comparison of the virulence genes cluster of lineage I, II and III

**Figure 2:**
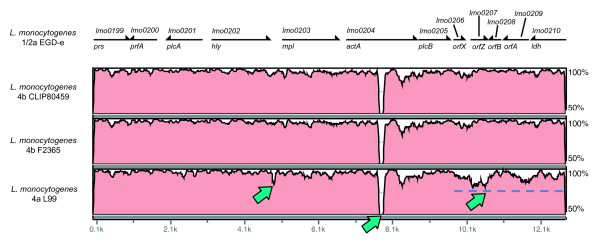
**Alignment of the virulence gene cluster of representatives of three*L. monocytogenes*lineages***L. monocytogenes* 1/2a EGD-e was used as reference genome. Nucleotide sequence identity of compared genomes is visualized. The top panel indicates location and direction of virulence genes.

All genes of the virulence gene cluster are present in the four studied strains [27]. We performed a nucleotide sequence alignment of the entire virulence genes cluster, using the EGD-e sequence as a reference. As shown in Figure 2 we identified a truncation in the *actA* sequence of the 4b and the 4a genomes. In addition, a small truncation upstream the *mpl* gene and a truncation of a short repeat region distal to the PrfA binding box of *mpl* was present in the 4a genome. However, the PrfA binding site was not affected. Moreover, the alignment identity decreased slightly in the latter half of the cluster, with differences most prominently visible in the regions containing *lmo0207* and *lmo0209*. *lmo0207* encodes a lipoprotein and was identified as one of the most divergent genes of the LIPI-1 when comparing three lineages.

Interestingly, both the *L. monocytogenes* 4b strains (CLIP80459 and F2365), and the *L. monocytogenes* 4a L99 strain, have an identical repeat truncation in the ActA protein compared to ActA of the 1/2a EGD-e (Additional file 2: Table S 2 Additional file 3: Table S 3). Such truncations in *actA* have been reported previously for strain 4a L99 and affect the speed of movement of intracellular bacteria [28]. We surveyed sequenced *act*A alleles present in GenBank and discovered that the truncation in the ActA protein is far more frequent in 1/2b and 4b strains (77% and 51% respectively) than in 1/2a strains (7.5%).

### Loss of surface proteins in lineage III

Several genes encoding internalin-like proteins are absent in the *L. monocytogenes* 4a L99 genome in comparison to the 1/2a EGD-e and the 4b strains (Additional file 4: Table S 4) as previously reported for lineage III strains [27,29]. The entire *inlGHE* cluster [30] is absent in the 4a L99 genome (Additional file 5: Table S 5) [27,30]. The corresponding loci in both 4b genomes are identical to each other, but different to strain 1/2a EGD-e. Another PrfA-independent internalin (InlJ) that has been shown to be specifically expressed only in vivo [31] is also absent from the 4a L99 genome. Similarly, Internalin C [27], involved in cell-to-cell spread and innate immune response in the vertebrate host [32-35], is absent in 4a L99 but is conserved in both 4b strains and 1/2a EGD-e. A comparable situation was identified for internalin F [27], however deletion mutants have not been shown to be reduced in invasion into non-phagocytic cells [36]. Apart from the absence of these characterized internalin genes, several other internalin-like genes (*lmo1666lmo2470* and *lmo2821*, Additional file 4: Table S 4) are present in the 1/2a EGD-e and 4b genomes, but are absent from the 4a L99 genome. In addition, we analysed the repertoire of genes encoding surface proteins for recently published 4a genomes of strain HCC23 [37] and M7 [38] as well as 4c FSL J2-071 (*Listeria monocytogenes* Sequencing Project, Broad Institute of Harvard and MIT; http://www.broad.mit.edu) (Additional files 4: Table S 4, Additional file 6: Table S 6 Additional file 7: Table S 7 Additional file 8: Table S 8 Additional file 9: Table S 9 Additional file 10: Table S 10 and Additional file 11: Figure S1). We confirmed by comparative genomics that these 4a genomes lack a similar number of surface proteins (Additional files 4: Table S 4, Additional file 6: Table S 6 Additional file 7: Table S 7 Additional file 8: Table S 8 Additional file 9: Table S 9 Additional file 10: Table S 10 and Additional file 11: Figure S 1). These findings were independently verified by additional PCR analysis to confirm the absence of genes encoding surface proteins for four 4a strains and three 4c strains, respectively. Half of the inspected chromosomal loci differed by PCR analysis among 4a and 4c genomes (Additional file 11: Figure S 1). Some non-internalin like cell-wall proteins that have been shown to be important for invasion are also absent, e.g. *auto* a GW-motif containing (Additional file 6: Table S 6), PrfA-independent, surface autolysin. Previous studies revealed an essential role for *auto* in the entry into non-phagocytic eukaryotic cells [39]. The *vip* gene product, a PrfA-dependent LPXTG protein (Additional file 7: Table S 7), described as a receptor for the eukaryotic Gp96 surface protein and important for late stages of infection [40], is also absent from the 4a L99 genome. In addition to these missing genes, InlI is slightly truncated. However Ami (Additional file 6: Table S 6), an important listerial adhesion protein seems to be present in a shorter version in both 4b strains [41,42], whereas the number of lipoproteins (Additional file 8: Table S 8), LysM- and (Additional file 9: Table S 9) NLPC/P60-motif containing proteins (Additional file 10: Table S 10) was comparable among the four strains under study.

Overall, in comparison to 1/2a EGD-e and the two 4b genomes, 4a L99 strain has lost a number of crucial determinants required for listerial invasion. The selective loss of genes primarily responsible for the first steps of infection may contribute to the poor invasion ability and the attenuated nature of the 4a L99 strain.

### Decay of phage genes in the *L. Monocytogenes* 4a L99 strain

The 1/2a EGD-e genome contains 79 prophage genes in two different loci, the 4a L99 genome includes 193 phage genes at four loci, while the 4b genomes encode with 16, for the smallest number of prophage genes limited to a single locus (also called the monocin-locus) at the same position in the chromosomes.

This monocin locus, a cryptic prophage region, is conserved in all *L. monocytogenes* lineages and includes the *lma* genes [43]. Although previously thought to be specific to *L. monocytogenes*, it was shown that *lmaDCBA* is also present in several apathogenic *L. innocua* strains. However, not all genes of the operon are present in all *L. monocytogenes* strains. The 4a L99 genome lacks *lmaA* and *lmaB* (Additional file 12: Figure S 2). The entire locus in 1/2a EGD-e and the two 4b genomes has 16 genes, but only five of these genes are present in the 4a L99 genome. *lmaA* and *lmaB* are absent in *L. welshimeri*. Interestingly, the structure of this prophage locus in strain 4a L99 and other lineage III strains is more similar to *L. welshimeri* than to other pathogenic listeriae (Additional file 12: Figure S 2).

### The CRISPR system of *Listeria*

**Figure 3:**
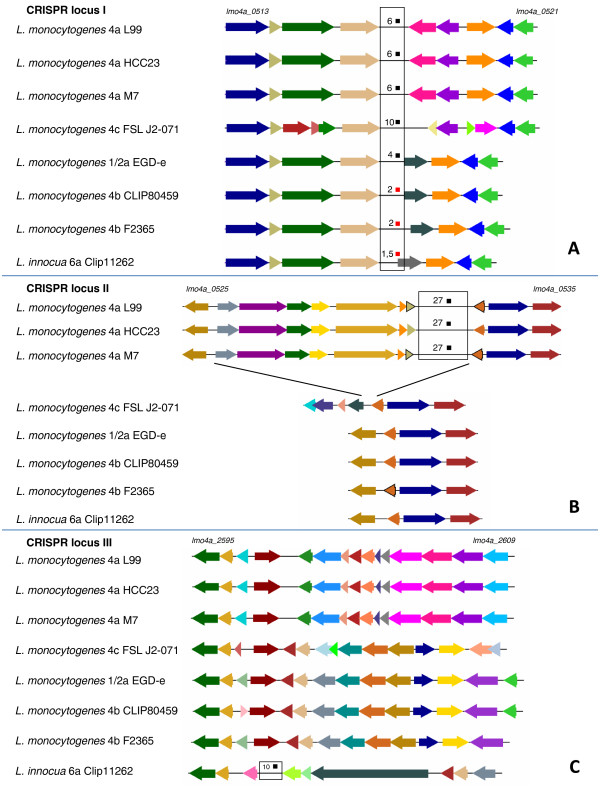
**Overview of CRISPR (clustered regularly interspaced short palindromic repeats) loci in*L****monocytogenes* 1/2a EGD-e, *L. monocytogenes* 4a L99, *L. monocytogenes* 4a HCC23, *L. monocytogenes* 4a M7, *L. monocytogenes* 4c FSL J2-071, *L. monocytogenes* 4b CLIP80459, *L. monocytogenes* 4b F2365 and *L. innocua* 6a CLIP11262*.* (A): CRISPR locus I is shown for all five listeriae, black boxes indicate complete CRISPR repeats, red boxes represent incomplete or truncated (*) CRISPR repeats. No *cas* genes were found to be associated with this locus. Flanking genes are conserved in 1/2a EGD-e and both 4b genomes. Comparison of the intergenic sequences with the 4a L99 genome revealed a sequence footprint of decaying repeat elements (2 repeat copies in both 4b genomes, and 1 copy in *L. innocua 6a* CLIP11262), indicating loss of the CRISPR repeats. (B): Locus II shows 29 copies of repeats and is associated with several *cas* genes (*cas2*, *cas3*, *cas5* and *cas6*. *cas1* is partially detectable, but seems to be truncated. (C): *L. innocua* 6a CLIP11262 harbours the CRISPR locus III at position 2.77 Mb in the genome, which is neighboured by a single *cas2* gene. No other CRISPR repeats nor any *cas* gene homologs were found in the 4b genomes.

The *L. monocytogenes* 4a L99 genome was found to contain two adjacent CRISPR loci (I and II) with CRISPR repeats (Figure 3A and 3B). Both loci contain sequences of length 35 bp separated by repeat sequences of length 29 bp. However, they differ considerably in the number of repeat copies (6 in locus I, and 29 in locus II, respectively). While locus I is highly conserved in the 4b strains, 1/2a EGD-e and *L. innocua*, locus II was exclusively present in 4a genomes of L99, HCC23, M7, but not in another lineage III genome of 4c FSL J2-071 (Figure 3 A-C). It is not known whether the CRISPR system is functional in the 4a L99 genome. However, by sequence similarity searches using the spacers to detect possible prophage DNA traces, we were able to identify the PSA prophage that is known to infect serotype 4 strains. Assuming a functional CRISPR system in 4a L99 suggests a resistance to the PSA bacteriophage (Additional file 13: Figure S 3).

### Gene duplications in the *Listeria* genomes expand metabolic systems

We found substantial evidence for a minimum of 231 to a maximum of 296 gene duplications in the *Listeria* genomes (Additional file 14: Figure S 4 and Additional file 15: Figure S 5). It is evident that the majority of these duplications are ancient events as they are shared among all species and the number of gene pairs with a very high percentage identity is very low (1-12% per strain). Functional classification of the duplicated genes revealed that many of these have important implications in metabolic pathways, like the pentose phosphate pathway, fructose and mannose metabolism, carbon fixation, glycolysis and pyruvate metabolism.

While several duplicated genes could be mapped to central metabolic pathways from the KEGG database, this was not possible for horizontally transferred genes (Additional file 16 Figure S 6 and Additional file 17: Figure S 7). However, not all duplicated genes seem to have arisen from true duplications, but some may have been transferred horizontally, like some PTS system genes that are *L. monocytogenes* EGD-e strain-specific genes. The number of genes classified into known metabolic pathways or systems was significantly higher for duplicated genes, while several horizontally transferred genes could not be mapped.

### Comparative intracellular transcriptomics of four *L. Monocytogenes* strains of the three major lineages

Comparative transcriptome analysis of *Listeria monocytogenes* strains of the two major lineages revealed differences in virulence, cell wall, and stress response [44]. Here we performed intracellular gene expression analyses using whole genome microarrays between four *L. monocytogenes* strains belonging to the three major lineages to investigate eventual differences. P388D1 murine macrophages were infected and total RNA was isolated four hours post infection and hybridized to bioarrays.

In order to determine the core intracellular response of *L. monocytogenes* we created a dataset of core-syntenic homologous genes for all four genomes and the expression data for these genes were compared. We found that in all strains studied the entire virulence genes cluster, (*prfA*, *plcA*, *hly*, *mpl*, *actA*, *plcB* and *orfX)* was highly induced within the infected host cells. Furthermore genes known to be important for bacterial survival, such as *hpt*, *clpE*, *bilEA* and two LRR domain-containing proteins (*lmo0514* and *lmo2445*) were upregulated in all strains.

Interestingly, three mannose transporting PTS systems (*lmo0021-lmo0024*, *lmo0781-lmo0784*, *lmo1997-lmo2002*), two fructose specific systems (*lmo2335* and *lmo2733*), two galacitol specific systems (*lmo0503*, *lmo0507*, *lmo0508* and *lmo2665-lmo2667*), two beta-glucoside systems (the partial system *lmo0373-lmo0374* and *lmo0874-lmo0876*), and two cellobiose specific systems (the partial system *lmo0901* and *lmo0914*-*lmo0916*) were commonly upregulated in all strains. These possibly represent the most frequently used substrates of listeriae in the cytosol. Only one mannose specific PTS system, (*lmo0096*-*lmo0098*) is downregulated by all studied strains (Additional file 18 Figure S 8 and Additional file 19: Text S 1).

**Figure 4:**
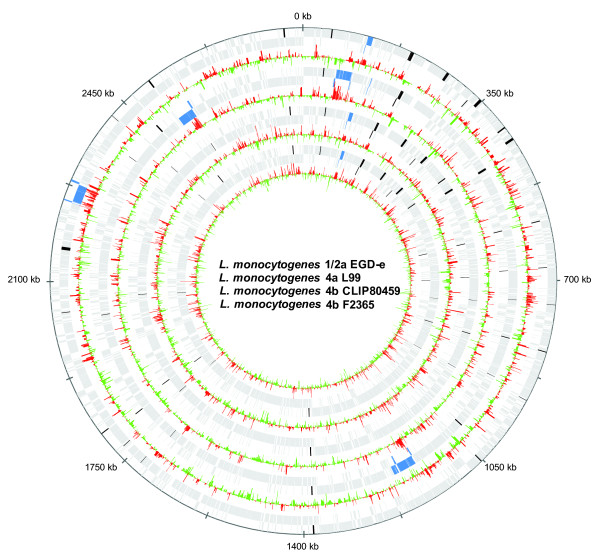
**Comparative transcriptomics of four*L. monocytogenes*genomes**: *L. monocytogenes* 1/2a EGD-e, *L. monocytogenes* 4a L99, *L. monocytogenes* 4b CLIP80459, *L. monocytogenes* 4b F2365 (from outside to inside). There are two tracks per strain: the first one shows the coding sequences (gray), phage genes (blue) and virulence genes (black). The second one visualizes increase (red) or decrease (green) of intracellular gene expression (log fold changes). Phage and virulence genes are clearly upregulated intracellularly. Data were illustrated using GenomeViz [26].

Most surprisingly, all *Listeria* strains studied expressed the genes of the *lma* operon and surrounding prophage genes of the monocin locus, including a conserved holin (*lmo0112lmo0113lmo0115lmo0116lmo0128*) during intracellular growth. However, the functions of several of these genes are not defined. The only locus that is conserved in all three lineages (albeit with some deletions in 4a L99) is the monocin *lma* locus. The *lmaA* gene product has been shown to provoke a delayed type hypersensitivity reaction in mice immune to *L. monocytogenes*. It is also secreted at 20°C but much less [45] at 37°C. The *lma* operon produces two transcripts, a 2100 bp *lmaDCBA* transcript expressed both at 20°C and 37°C, and a 1050 bp *lmaBA* transcript induced at lower temperatures [43]. Additional prophage genes were highly expressed in the individual strains (Figure 4). Taken together, high intracellular prophage gene expression, despite several differences in prophage gene content, is one of the most striking observations across all *Listeria* lineages.

All strains showed induction of the *eut* operon suggesting that ethanolamine may be used as a carbon and nitrogen source in intracellular conditions. The zinc transporters were also commonly upregulated indicating a role of zinc in intracellular survival as well as the spermidine/putrescine ABC transporters (*potB, potC* and *potD*). Furthermore, the non-oxidative branch of the pentose phosphate pathway was utilized by all listeriae, possibly to generate NADPH for countering oxidative stress in intracellular conditions. The upregulation of genes of the pentose phosphate pathway has been shown previously [19,20,46] and it has been speculated that it is important for generation of erythrose-4-phosphate for aromatic amino acid biosynthesis or for generation of pentose sugars. Accordingly; we observed a downregulation of several genes involved in pyrimidine and purine biosynthesis from pentose sugars (e.g. *lmo1463lmo1497lmo1565lmo1832lmo1836lmo1856lmo1929lmo2154lmo2155lmo2390* and *lmo2559*).

Downregulated genes included the *agr* locus (*lmo0048-lmo0051*) as demonstrated previously [20,46] and several genes of the tryptophan biosynthesis operon (*trpAtrpBtrpF* and *trpD*), and some tRNA synthetase genes (*ileSvalSglyS* and *glyQ*). Diminished energy generation was indicated by decreased expression of the cytochrome genes cluster *cytABCD*. With respect to the pentose phosphate pathway, we detected downregulation of the phosphoribosyl pyrophosphate synthetase (*prs, lmo0199*) gene, which is required for the production of PRPP (phosphoribosyl pyrophosphate) that links the pentose phosphate pathway to the biosynthesis of purines and pyrimidines. While several genes of the glycolytic operon, and several individual genes were downregulated by 1/2a EGD-e, 4b CLIP80459 strain or 4a L99, the 4b F2365 strain showed increased expression (Additional file 20: Text S 2).

### Differences in flagellin expression are the most prominent differences among strains

To address the observation that strain 4b CLIP80459 grows more efficiently inside the host than strain 4b F2365, we performed a direct comparison of the transcriptome data derived from these two strains. Most important differences were found in the regulation of flagellar genes. While intracellular bacteria of strain 4b F2365 upregulated a substantial number of flagellar genes, including *fliS*, *fliI*, *flhA*, *fliF*, *filE*, *flgB*, *flgC*, *flgG*, *fliD* as well as the transcriptional regulator *degU* (*lmo2515*), in the 4b strain CLIP80459 only *fliR* was upregulated. When comparing the intracellular transcriptome of strain 4a L99 to the 1/2a and 4b strains the most striking difference was again the expression of the flagellar operon. We observed a strong induction of nearly all flagellar genes in the operon, including flagellin (Additional file 21: Text S 3) (homologues of *lmo0675*, *lmo0676*, *lmo0681*, *lmo0685*, *lmo0686*, *lmo0690*-*lmo0696*, *lmo0698*-*lmo0701*, *lmo0703*-*lmo0706*, *lmo0708*, *lmo0709*, *lmo0712*, *lmo0714* and *lmo0715*) in strain 4a L99. Strong expression of these genes is counterproductive within infected cells, because it probably enables the host to efficiently detect bacterial presence and the formation of an inflammasome.

Apart from genes that are important for pathogen recognition mechanisms by the host, a concerted expression profile (Additional file 22: Figure S 9) involving genes of cell wall synthesis, host cell invasion, response to oxidative stress, utilization of host carbohydrates and propanediol, which are crucial for intracellular survival as well as virulence and surface proteins were identified.

### Differential growth of the three lineages and ∆*lmaB* and ∆*lmaD* isogenic mutants in a mouse infection and cell infection models

We observed a severe deficiency in entry of strain L99 in HeLa and Caco-2 cells as well as poor cell-to-cell transmission with macrophages and L929 fibroblasts when compared to 1/2a EGD-e (data not shown). Impaired invasion ability of host cells may be due to lack of several internalin genes in the genome of strain 4a L99. It is likely that both, decreased invasive ability and strong intracellular expression of flagellar genes contribute towards the rapid clearance of the 4a L99 strain in in vivo experiments in mice. Upregulation of several DNA repair genes was also seen in strain 4a L99 compared to the other strains, e.g. (*recF*, *recN*, *radA* and *mutL*), suggesting genomic damage during the infection process.

**Figure 5:**
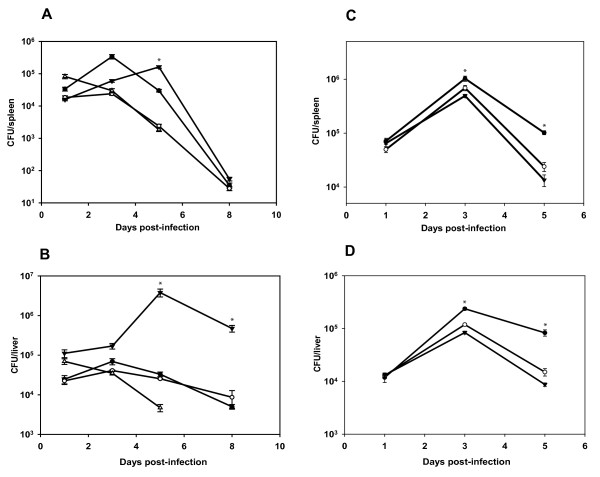
**Murine infection studies with three different*Listeria*serotypes and two chromosomal deletion mutants of ∆*lmaB*and ∆*lmaD*of*L. monocytogenes*1/2a EGD-e** Mice were infected i.v. with 2000 cfu of *L. monocytogenes* serotypes 1/2a EGD-e (filled circles), 4b F2365 (open circles), 4b CLIP80459 (filled triangles), and 4a L99 (open triangles). On days 1, 3, 5, and 8 after infection, the numbers of viable bacteria in spleens (A) and livers (B) of three animals per group were determined (*P* ≤0,05 and *P* ≤0,001 of 4b CLIP80459 vs. 1/2a EGD-e and 4b F2365 vs. 1/2a EGD-e in spleen and liver respectively). Bacterial load in mice organs were also determined following i.v. infection with 2000 cfu of *L. monocytogenes* 1/2a EGD-e wild type strain (filled circles) as well as its isogenic mutants ∆*lmaB* (open circles), and ∆*lmaD* (filled triangles). On days 1, 3, and 5 after infection, the numbers of viable bacteria in spleens (C) and livers (D) of three animals per group were determined (*P* ≤0,05 and *P* ≤0,01 of 1/2a EGD-e versus ∆*lmaB* and ∆*lmaD* in spleen and liver respectively). Data presented are representative of three independent experiments. An asterisk indicates means that are significantly different from the wild type. Significance analysis was performed with student *t*-test.

To further assess the virulence potential of the three lineages, we performed mouse infection experiments with each of the four strains (1600 cfu/mouse), and measured bacterial loads in spleens and livers at different time points (Figure 5A and 5B). The 4a L99 strain was cleared rapidly from the mice and was not detectable after five days of infection, in accordance with previous results [18], indicating that the 4a L99 strain is attenuated in its pathogenicity. However, the other three strains were able to survive in both spleens and livers of infected mice. Interestingly, while they could comparably replicate in the spleen, the 1/2a EGD-e and the 4b F2365 bacterial loads in liver were significantly lower than the 4b CLIP80459 strain whose counts remained significantly higher even on days five and eight post-infection. Isogenic mutants of Δ*lmaB* and Δ*lmaD* showed similar counts in mice spleens and livers. However, both mutants have shown a significantly lower level of growth than 1/2a EGD-e on days 3 and 5 post-infection (Figure 5C and 5D).

## Discussion

We sequenced and analysed the genomes of representatives of three major lineages of species *L. monocytogenes* to correlate gene content with (i) its wide spectrum of pathogenic abilities, (ii) its differing properties for survival in the hosts, and (iii) its adaptive properties during growth under extracellular conditions.

### Decay of surface proteins in the virulence attenuated *L. Monocytogenes* 4a strain

Analysis of the 4a L99 genome revealed extensive loss of a large number of internalins, internalin-like proteins and other surface proteins important for invasive ability. For strain 4a L99, which was isolated from contaminated food in the 1950’s, it might be possible that mutations have taken place over this lengthy time of storage under in vitro conditions. Surprisingly, a previously known *act*A truncation in the 4a genomes of L99, HCC23 and M7, was also found in a higher number of lineages I strains compared to lineage II, but not in the *act*A gene of another lineage III strain of 4c FSL J2-071 indicating a serotype-specific heterogeneity of ActA sequences within the genus *Listeria*. The loss of this proline-repeat in ActA is correlated with lowered actin-based motility in the cytosol. In addition, comparative nucleotide analysis indicated that the latter half of the LIPI-I pathogenicity island in strain 4a L99 has diverged significantly from that of the 4b and 1/2a strain leading to a loss of the open reading frames *lmo0206* to *lmo0209*. Loss of *lmo0206* (*orfX*) has been shown to confer a severe growth effect on survival in macrophages, [20] while loss of *lmo0207* has a small effect on growth in macrophages and no data are presently available for *lmo0208* and *lmo0209* and their role in virulence.

### Differential regulation of intracellular flagella gene expression by strains of different lineages

Highly sensitive and widely distributed host microbe-associated microbial pattern receptors (TLRs and NLRs) continuously patrol the cell surface, endosomes and the cytosol for signs of microbial presence by sensing cell wall components, bacterial DNA, lipoproteins and flagellin. Ligands may be shared between the surface and the cytosolic receptors, e.g. cell wall components and flagellin may be sensed both by TLRs and also by cytosolic receptors. We detected the intracellular expression of the flagellin gene in 1/2a EGD-e [20]. Recently, it has been shown that cytosolic flagellin, expressed by *L. monocytogenes* strain 10403 S (serotype 1/2a) is detected by multiple Nod-like receptors, including IPAF and NALP3, and also by a pathway involving the adaptor protein ASC and the cytosolic DNA sensor AIM2, which is required for the formation of the inflammasome [47-49]. Detection of flagellin in the cytosol via these pathways leads to caspase-1 mediated cleavage of pro-IL-1B and release of active IL-1B. Mice lacking caspase-1 or ASC are unable to mount active IL-1B response to intracellular pathogens such as *Shigella flexneri* and *Francisella tularensis*[50,51]. All strains investigated in this study were found to express flagellar genes in the cytosol, except for strain 4b CLIP80459. The ability to successfully downregulate flagellar (*flaA*) gene expression is probably critical for evading host detection and promoting bacterial intracellular growth. In line with this observation, a 1/2a EGD-e chromosomal deletion mutant of the gene displayed increased survival in mouse infection assays [52].

In keeping with this finding, both strains 4b F2365 and 4a L99 displayed strong induction of several flagellar genes during intracellular growth and were more readily cleared from the host. This suggests strain-specific differences in the ability to avoid host recognition can lead to large differences in virulence manifestation, despite several commonalities in the adaptations of the lineages to the intracellular lifestyle. Although all the strains investigated in this study were able to induce all genes of the virulence genes cluster intracellularly, it is likely that there are a multitude of effects including differences in virulence gene expression, uptake of carbohydrates, membrane protein expression and flagellar biosynthesis, all of which contribute to the observed phenotypic properties.

### Effects of gene duplication events on metabolic adaptation and survival within the host

The processes of gene duplications, horizontal gene transfer and gene loss influence the short- and long-term evolution of prokaryotic genomes. The benefits of gene duplications in the short term can be seen clearly in conditions of antibiotic treatment [53,54], toxin exposure [55], heavy metal stress [56,57], extreme temperatures [58], nutrient limitation [59,60] and even parasitic and symbiotic lifestyles [54,61]. Duplications found in all *Listeria* genomes seem to have been ancient i.e. precede species differentiation, with only the exception of the recent prophage duplication in *L. innocua* 6a CLIP11262*.* Classification of duplicated genes revealed several paralogous genes in metabolic pathways, while very few horizontally transferred genes could be classified at all.

The highest numbers of gene duplications were identified in the following categories: ABC transporters, PTS systems, pentose phosphate pathway, starch and sucrose metabolism, fructose and mannose metabolism, and carbon fixation. Surprisingly, we found a high number of duplicated gene paralogues involved in the regulation of the non-oxidative branch of the pentose phosphate pathway and in the generation of ribose-5-phospate from ribulose-5-phosphate. Under conditions of intracellular growth, we observed differences in the ability of the lineages to express horizontally transferred genes. 1/2a EGD-e was most successful in this regard (17 genes), followed by 4a L99 (10 genes), 4b F2365 (6 genes) and 4b CLIP80459 (2 genes). Apart from the horizontally transferred genes, differences in the expression of strain-specific genes in the cytosol were apparent (1/2a EGD-e: 45; 4a L99: 49; 4b F2365 11; 4b CLIP80459: 3).

PTS systems enable listeriae to utilize host carbohydrates, a mechanism that is essential for the intracellular survival. PTS systems (EII) for the utilization of fructose and beta-glucosides, mannose and cellobiose were most frequently observed in the investigated *Listeria* genomes. Although the numbers of PTS systems are comparable among the investigated genomes (Additional file 18: Figure S 8), even a slight difference in presence/absence of a PTS system available as an additional carbohydrate utilization mechanism may have dramatic effects on listerial survival inside the host cytosol [61-63], specifically on the master regulator PrfA [61,62,64,65]. For instance, the pentitol PTS system in 1/2a EGD-e is not present in either the 4b or the 4a L99 genomes. A transposon insertion mutant of this system (*lmo1971*) has been shown to have significantly attenuated growth in epithelial cells [46]. Several partial PTS systems are also present in the genome (Additional file 19: Text S 1). These are independently expressed intracellularly, and represent broadly shared and commonly regulated systems. In accordance, the pathogenic strain 4b CLIP80459 was found to upregulate more PTS systems than strain 4b F2365, which may contribute to better intracellular survival of 4b CLIP80459.

In addition to phosphorylated sugars, there are other nitrogen and carbon sources available to intracellular bacteria, such as ethanolamine. Ethanolamine is used as substrate and an energy supply by *Salmonella enterica* grown under anaerobic conditions and is suggested to be used by other bacteria [66]. A locus homologous to that of the ethanolamine operon of *S. enterica* has also been described in *Listeria*[67]. The gene organization of the locus is not identical to the *Salmonella* cluster, but all the genes of the cluster have homologous sequences in *Listeria* (Additional file 23: Figure S 10). Previous studies identified genes of the locus to be upregulated intracellularly during infection and were shown to play a critical role for intracellular survival [46]. Our data support this observation and further demonstrate upregulation of several genes of this locus across all three pathogenic lineages of *Listeria*, suggesting that the functions of the locus are conserved. However, since the locus is also present in the apathogenic *L. innocua* strain 6a CLIP11262, it may exemplify a general requirement of *Listeria* to cope with nutrient rather than a specific virulence adaptation. Furthermore, degradation of the phagosomal membrane that traps intracellular listeriae*,* results in the release of ethanolamine as a byproduct and may serve an energy source in the host cytosol.

Not only the efficient recruitment of carbohydrate substrates, but also the differential channeling through different pathways represents an important adaption within the host cytosol. It has been shown that an essential mechanism to counteract oxidative stress is to reroute carbohydrate flux via the pentose phosphate pathway, which is required for the biosynthesis of reductive substrates rather than through glycolysis pathway [68]. Indeed, we observed that all lineages prefer to channel carbohydrate flux via the pentose phosphate pathway, rather than glycolysis. In contrast to the other strains, only strain 4b F2365 was unable to downregulate glycolysis, suggesting that the inability to route sugars efficiently via pentose phosphate contributes to the poor intracellular growth of this strain.

### The CRISPR system in *Listeria* reveals expansion and atrophy

A CRISPR (Clustered, regularly interspaced short palindromic repeats) locus, associated with several *cas* genes was identified in the 4a L99 genome. CRISPRs are highly divergent loci found in genomes of all archaea and several bacteria [69]. A CRISPR system is composed of the *cas* (CRISPR-associated) genes, a leader sequence and arrays of direct repeats separated by non-repetitive spacer sequences resulting in a RNA-interference like innate phage-resistance mechanism [70]. A recent study in *Streptococcus thermophilus* demonstrated how bacteria are able to integrate new spacer sequences derived from infecting phages, directly into the CRISPR arrays, and that this ability confers phage-resistance [71]. The mechanism of resistance has also been elucidated [70]. Among the genomes compared in this study, only the 4a L99 genomes of L99, HCC23 and M7 possesses *cas* genes and several CRISPR repeats. There are only two repeats in each 4b genome, five in 1/2a EGD-e a single one in *L. innocua* 6a CLIP11262, but none of these strains harbour identifiable *cas* genes. In addition, a small sRNA *rliB* is located in the repeat region of 1/2a EGD-e and contributes to virulence in mice [72]. We were also able to detect a DNA sequence of a potential prophage (PSA) using the spacers from the 4a genome. As prophages evolve quite rapidly, it is likely that this acquisition is a recent event.

### Distinct role of intracellularly upregulated phage genes in virulence of listerial strains

The four *L. monocytogenes* strains have different numbers of prophage genes (1/2a EGD-e: 79; 4a L99: 191; 4b CLIP80459: 16 and 4b F2365: 16) distributed in different loci. Regardless of location and lineage, all strains expressed several prophage genes within the infected host cell. However, only a single locus, the *lma* locus is conserved across the three lineages and is also induced during infection. The role of prophage genes in the virulence of *Listeria* has not been examined in detail. We show that chromosomal deletion mutants of two genes in this locus (*lmaB* and *lmaD*) resulted in growth reduction of 1/2a EGD-e in a murine infection model. Although the underlying mechanisms leading to the attenuated phenotypes remain unclear, a recent study revealed that prophage diversification represents an essential mechanism for short-term genome evolution within the species *L. monocytogenes*[73,74] and is subject of further investigation.

## Conclusion

*Listeria monocytogenes* is arguably one of the best characterized pathogens and has been established as an unparalleled model microorganism in infection biology. Detailed understanding of differences in virulence of the three major lineages of *Listeria* provides us with invaluable information about evolutionary adaptation of this pathogen. Here we used comparative genomics and whole-genome based transcriptome analysis of strains from all lineages to obtain a comprehensive view as to how these strains have evolutionarily diverged. This approach suggests that (i) reductive evolution of strains of serotype 4a such as L99, HCC23 and M7 is the major force driving the attenuated phenotype, (ii) acquisition and adaptation of prophage genes and metabolic systems, respectively, identify novel virulence-associated factors of listeriae and (iii) listeriae avoid detection and subsequent immune response of the host via downregulation of surface structures and by differences in intracellular expression of flagellar genes.

## Methods

### Strains and growth conditions

Four *L. monocytogenes* strains were used in the study, *L. monocytogenes* 1/2a EGD-e [14], *L. monocytogenes* 4a L99 [18], *L. monocytogenes* 4b CLIP80459 [17], *L. monocytogenes* 4b F2365 [15] and chromosomal deletion mutants of *L. monocytogenes* 1/2a EGD-e Δ*lmaB* and Δ*lmaD*. Bacteria were grown in brain heart infusion (BHI) broth (Difco) at 37°C with shaking. For further comparative genomic analysis *L. monocytogenes* 4a HCC23 [37]*L. monocytogenes* 4a M7 [38] and *L. monocytogenes* 4c FSL J2-071) (*Listeria monocytogenes* Sequencing Project, Broad Institute of Harvard and MIT; http://www.broad.mit.edu) was used.

### Genome sequencing and annotation

In brief, genome sequencing *L. monocytogenes* 4a L99 was performed on ABI PRISM 3100 or 3730xl Genetic Analyzers (Applied Biosystems). Whole genome shotgun sequencing was performed by LGC (Berlin, Germany). Sequence data were analysed and assembled using Phred/Phrap/Consed [75,76]. A total number of 27,637 sequences of shotgun libraries, 1684 fosmid and 671 PCR gap closure sequences were assembled by the Phrap software resulting in a ~6.7-fold coverage. Genome annotation was performed as previously described [3].

Genome sequencing of *L. monocytogenes* 4b CLIP80459 was performed using the conventional whole genome shotgun strategy [77,78]. One library (2–3 kb inserts) was generated by random mechanical shearing of genomic DNA and cloning into pcDNA-2.1 (Life technologies) and recombinant plasmids were used as templates for cycle sequencing reactions. Samples were loaded on capillary automatic 3700 and 3730 DNA sequencers (Applied Biosystems). In an initial step 35,610 sequences were assembled into 361 contigs using the Phred/Phrap/Consed software [75,76]. CAAT-Box [79] was used to predict links between contigs. 379 PCR products amplified from *L. monocytogenes* CLIP80459 chromosomal DNA as template were used to fill gaps and to re-sequence low quality regions. Final assembly resulted in a ~7.8-fold coverage. Genome annotation was performed as previously described [14].

### Alignment of the virulence gene cluster

The alignment was performed using MAVID [80] after extracting the virulence gene cluster of all genomes. The plot was created using VISTA [81].

### ActA repeat analysis

Available ActA protein sequences for all *L. monocytogenes* strains were retrieved from GenBank (http://www.ncbi.nlm.nih.gov/Genbank/). Only sequences that contained at least 500 amino acids (reference strain 1/2a EGD-e ActA: 639 amino acids) were downloaded (774 sequences). It was possible to assign a lineage to only 386 ActA sequences. Duplicates with identical length, strain and sequence were also removed, leaving a total of 218 sequences for the analysis. These were aligned using ClustalW and the alignment of repeat regions was examined manually.

### Single nucleotide polymorphisms

Single nucleotide Polymorphisms (SNPs) were detected by the MUMmer [25] and SNPs were mapped to coding regions using PERL scripts. The SNP-density per gene normalized by gene length was calculated and the data were visualized in GenomeViz [26].

### CRISPR repeats analysis

Comparative visualization of the CRISPR related genome loci was performed by GECO [82]. CRISPR repeats were identified using the PILER-CR software [83]. Subsequent analysis and visualization of repeat footprints was performed using BLAST and ACT [84].

### Horizontal gene transfer and gene duplications

Horizontally transferred genes were detected using SIGI [85] and SIGI-HMM [86]. Duplicated genes were identified using BLAST cut-offs of at least 40% identity and 80% coverage considering both sequences.

### Cell culture and infection model

All cell culture experiments were performed as described by Chatterjee and colleges [20].

### Microarrays

For each of the four strains of the study, a genome-wide custom microarray chip was designed and implemented using the Geniom One platform from Febit Biomed GmbH, Germany. All transcriptome studies were performed with this platform. Complete details of the protocols are provided in the ArrayExpress database (http://www.ebi.ac.uk/microarray-as/ae/). Data were background corrected and then normalized using quantile normalization [87]. Pearson’s correlation coefficients were used to assess reproducibility within at least two technical and three biological replicates (r^2^ > =0.94 in all cases). The significance analysis of microarrays (SAM) program was used to analyze the data [88] as an unpaired response.

### Construction of the deletion mutants Δ*lmaB* and Δ*lmaD*

Chromosomal in frame deletion mutants of *L. monocytogenes* 1/2a EGD-e Δ*lmaB* and Δ*lmaD* were constructed by generating the 5′ (with primers P1 and P2) and the 3′ (with primers P3 and P4) flanking region of the gene concerned. Primers used to generate the flanking regions are shown (Additional file 24: Table S 11). The purified PCR fragments of 5′ and 3′ flanking regions were amplified using primer P1 and P4, ligated into pCRII (Life technologies) and transformed into *E. coli* InvαF’ electrocompetent cells (Life technologies). Subsequently, the vector was digested with restriction enzyme *Eco*RI and ligated into the temperature sensitive suicide vector pAUL-A which was digested with the same enzymes and transformed into *E. coli* InvαF’ electrocompetent cells. Plasmid DNA of pAUL-A bearing the fragment was isolated from the recombinants and used to transform *L. monocytogenes* EGD-e to generate the chromosomal deletion mutants as described in detail by Schaeferkordt et al. [89]. The deletion in the gene concerned was identified by PCR and confirmed by sequencing the PCR fragment using primers P1 and P4.

### Murine infection assay

Primary infection with *L. monocytogenes* serotypes and mutants was performed by intravenous injection of viable bacteria in a volume of 0.2 ml of PBS. Bacterial growth in spleens and livers was determined by plating 10-fold serial dilutions of organ homogenates on BHI after several days. The detection limit of this procedure was 10^2^ CFU per organ. Colonies were counted after 24 h of incubation at 37°C. Six- to eight-week-old female BALB/c mice, purchased from Harlan Winkelmann (Borchen, Germany), were used in all experiments.

### Ethics statement

This study was carried out in strict accordance with the regulation of the National Protection Animal Act (§7-9a Tierschutzgesetz). The protocol was approved by the local Committee on the Ethics of Animal Experiments (Regierungsbezirk Mittelhessen) and permission was given by the local authority (Regierungspraesidium Giessen, Permit Number: GI 15/5-Nr.63/2007).

### Statistical data analysis of infection experiments

All infection experiments were performed a minimum of three times. Significant differences between two values were compared with a paired Student’s *t*-test. Values were considered significantly different when the *p* value was less than 0.05 (*p* < 0.05).

### Nucleotide sequence and microarray accession number

The genome sequences have been deposited in the EMBL database with accession numbers FM211688 for *L. monocytogenes* 4a L99 and FM242711 for *L. monocytogenes* 4b CLIP80459 respectively. The microarray data have been submitted to ArrayExpress with the accession number E-MEXP-1947.

## Competing interests

The authors declare that they have no competing interests.

## Author’ contributions

TH and TC designed the study. CB, PG, CS and TH performed genome sequencing, MAM isolated total RNA. SV performed microarray samples preparation and hybridization. TH, AB, CTK UK, ED, BI, RG, SS, PG, CR, CB, AG, SO and WM performed the genome annotation and analysis work. RG, AB, CTK, BI, TH and TC drafted and wrote the manuscript. All authors contributed to and approved the final manuscript.

## Supplementary Material

Additional file 1 **Table S1.** Nucleotide analysis of *actA* repeats of *Listeria*. Click here for file

Additionaf file 2 **Table S2.** Prediction of LRR region containing proteins by Augur [90]. Click here for file

Additional file 3 **Table S2.** Prediction of proteins containing GW modules by Augur [90]. Click here for file

Additional file 4 **Table S4.** Prediction of LPXTG motif harbouring proteins by Augur [90]. Click here for file

Additional file 5 **Table S5.** Prediction of lipoproteins by Augur [90]. Click here for file

Additional file 6 **Table S6.** Prediction of LysM domain containing proteins by Augur [90]. Click here for file

Additional file 7 **Table S7.** Prediction of NLPC/P60 domain containing proteins by Augur [90]. Click here for file

Additional file 8 **Table S8.** Comparative CRISPR analysis table. Click here for file

Additional file 9 **Table S9.** Metabolosome of *L. monocytogenes* 1/2a EGD-e. Click here for file

Additional file 10 **Table S10.** Primers used in this study. Click here for file

Additional file 11 **Figure S1.** Comparative analysis of *L. monocytogenes* ActA protein sequences. Click here for file

Additional file 12 **Figure S2.** Comparison of the *inlGHE* locus in the three listerial lineages. All three genes in this cluster have been absent in the *L. monocytogenes* 4a L99 genome. Click here for file

Additional file 13 **Figure S3.** Genome analysis of *lmaDCBA* region of six listeriae. Comparative analysis was performed using GECO [82] applying bidirectional pairs. Click here for file

Additional file 14 **Figure S4.** Frequency of distributions of the percentage identity between all duplicated gene pairs in the *Listeria* genomes. Click here for file

Additional file 15 **Figure S5.** Gene duplication and horizontal gene transfer in *Listeria* genomes. Click here for file

Additional file 16 **Figure S6.** Duplication vs. HGT classifiable genes in listeriae. Click here for file

Additional file 17 **Figure S7.** Complete PTS Systems in *L. monocytogenes* strains. Click here for file

Additional file 18 **Figure S8.** Partial PTS Systems in *L. monocytogenes* strains. Click here for file

Additional file 19 **Text S1.** SNP analysis of three listerial lineages. Click here for file

Additional file 20 **Text S2.** Differential regulation of glycolysis in *L. monocytogenes* 4b F2365. Click here for file

Additional file 21 **Text S3.** Comparison of two *L. monocytogenes* 4b strains CLIP80459 and F2365. Click here for file

Additional file 22 **Figure S9.** Confirmation of lacking genes encoding surface proteins in four *L. monocytogenes* 4a strains and three *L. monocytogenes* 4c strain generated by PCR analysis. Click here for file

Additional file 23 **Figure S10.** Intracellular flagellin expression data of *L. monocytogenes* 1/2a EGD-e, *L. monocytogenes* 4a L99, *L. monocytogenes* 4b CLIP80459 and *L. monocytogenes* 4b F2365 generated by qRT-PCR analysis. Click here for file

Additional file 24 **Figure S11.** List of gene duplication in *Listeria* genomes. Click here for file
