# Supplementary material for: Comparative genomics and transcriptomics of lineages I, II, and III strains of Listeria monocytogenes
Source: BMC Genomics. 2012 Apr 24;13:144. doi: 10.1186/1471-2164-13-144 (PMC3464598; doi:10.1186/1471-2164-13-144)
Supplement: Additional file 1 — Table S1. Nucleotide analysis of actA repeats of Listeria. [file 1471-2164-13-144-S1.pdf]

SNP analysis of three listerial lineages

lm4b_01815, a putative peptidoglycan bound protein, was absent in the 4b F2365 genome. However, this protein is present in other *Listeria* genomes, even in apathogenic *L. innocua* 6a CLIP11262, while being highly divergent (amino acid sequence identity <30%). However, apart from these surface proteins, a few regulatory proteins were also identified as divergent, e.g. *Lm4b_00382* (similar to *Salmonella typhimurium* peptidase E) and its putative regulatory protein *Lm4b_00384*, both present in all *Listeria* examined here. In addition, some ABC transporters and metabolic genes, along with several hypothetical genes were identified. There seemed to be no particular overrepresentation of any kind of cellular pathway in these genes, indicating that several different pathways may be subject to selective pressures at different levels at the same time in the divergence of these two 4b genomes.

The comparison of the 4a L99 genome with 1/2a EGD-e also reflected the larger evolutionary divergence between the 1/2a EGD-e and the 4a L99 genomes as compared to the divergence between the two 4b genomes as the number of SNPs per gene length was much higher for several genes than in the 4b genomes. Interestingly, *lmo2549* was identified in this comparison. This gene is a cell wall associated teichoic acids glycosylation protein that may have direct impact on the antigenic-structure of the listerial cell walls. As in the comparison of both 4b strains, some important surface proteins, e.g. *lmo1799* (LPXTG motif containing protein) and an autolysin (*lmo1215*) were also identified. Apart from these genes a large number of hypothetical genes, as well as some phage genes (that are shared by both genomes) were indicated. The comparison of the 1/2a EGD-e genome to both 4b genomes revealed quite similar genes. Once again, several hypothetical proteins, along with some surface proteins e.g. *lmo0409* (internalin F), *lmo2552* (*murZ*) and *lmo2549* (*gtcA*) were identified.

To identify genes that are most divergent in the three lineages, another comparison was performed between three genomes (1/2a EGD-e, 4a L99 and 4b CLIP80459). Orthologous genes across all three genomes were aligned to identify the most divergent orthologous gene

groups. Classification of 156 orthologs into COG categories that have less than 90% alignment identities revealed that the largest number of genes belong to the categories of amino-acid transport and metabolism (*lmo0363*, *lmo0561*, *lmo0758, lmo1259, lmo1260, lmo1387*, *lmo1589*, *lmo1988*, *lmo2022*, *lmo2043* and *lmo2777*), DNA replication, recombination and repair (*lmo0660, lmo0996, lmo1273*, *lmo1274*, *lmo1404*, *lmo1484*, *lmo1549*, *lmo1942*, *lmo1975* and *lmo2513*) and transcription (*lmo0430*, *lmo0436*, *lmo0976*, *lmo1126*, *lmo1134*, *lmo1405*, *lmo2146*, *lmo2513* and *lmo2551*). In addition, six lipoproteins (*lmo0207*, *lmo0617*, *lmo1073*, *lmo1264*, *lmo1265* and *lmo2080*) and some LPXTG proteins (*lmo1290*, *lmo1413* and *lmo1746*) were also identified.
