# Supplementary material for: Comparative genomics and transcriptomics of lineages I, II, and III strains of Listeria monocytogenes
Source: BMC Genomics. 2012 Apr 24;13:144. doi: 10.1186/1471-2164-13-144 (PMC3464598; doi:10.1186/1471-2164-13-144)
Supplement: Additionaf file 2 — Table S2. Prediction of LRR region containing proteins by Augur [90]. [file 1471-2164-13-144-S2.pdf]

Lineage	Serotype	Total actA sequences	Number of truncated sequences	Percentage of truncated sequences [%]
I	1/2b	81	63	77,78
I	4b	54	28	51,85
II	1/2a	40	3	7,5
III	4a	4	4	100
III	4c	1	0	0
