# Supplementary figures and images for: Comparative genomics and transcriptomics of lineages I, II, and III strains of Listeria monocytogenes
Source: BMC Genomics. 2012 Apr 24;13:144. doi: 10.1186/1471-2164-13-144 (PMC3464598; doi:10.1186/1471-2164-13-144)

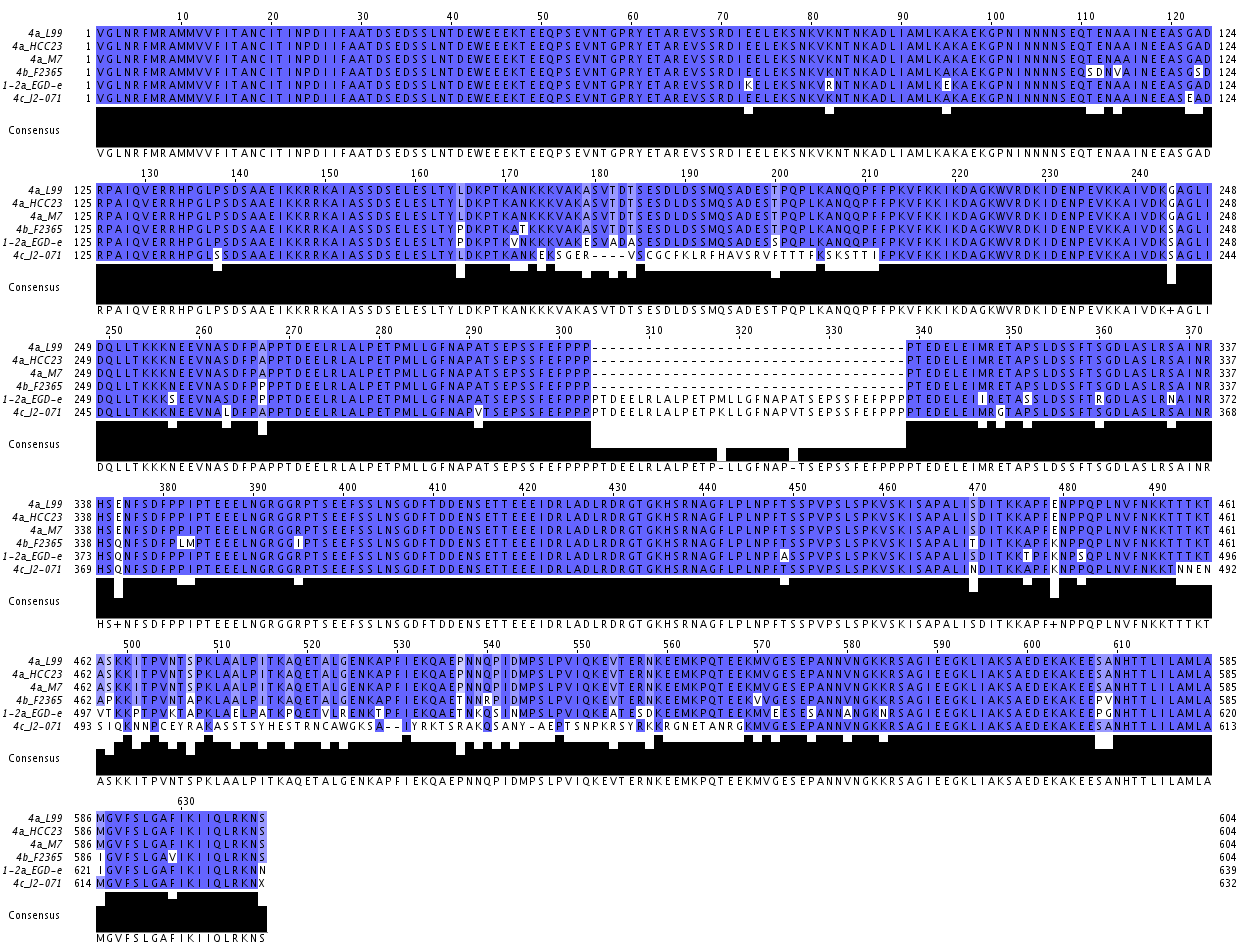

Supplement: Additional file 3 — Table S2. Prediction of proteins containing GW modules by Augur [90]. [file 1471-2164-13-144-S3.png]

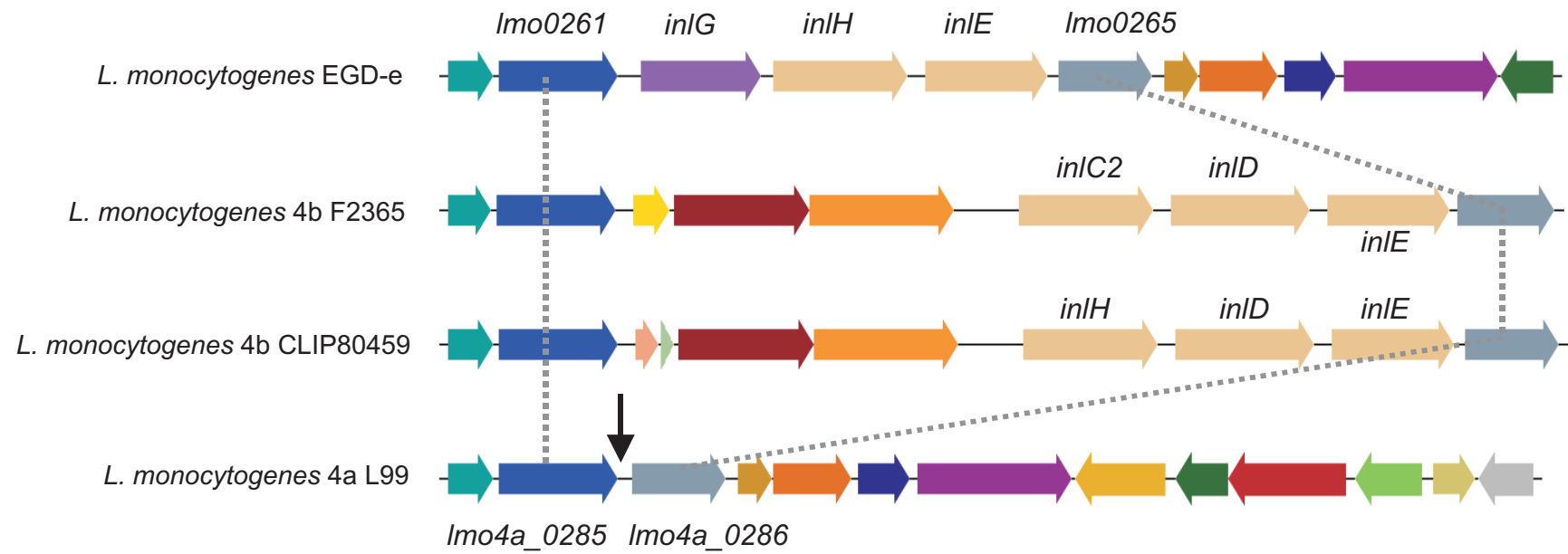

Supplement: Additional file 5 — Table S5. Prediction of lipoproteins by Augur [90]. [file 1471-2164-13-144-S5.pdf]

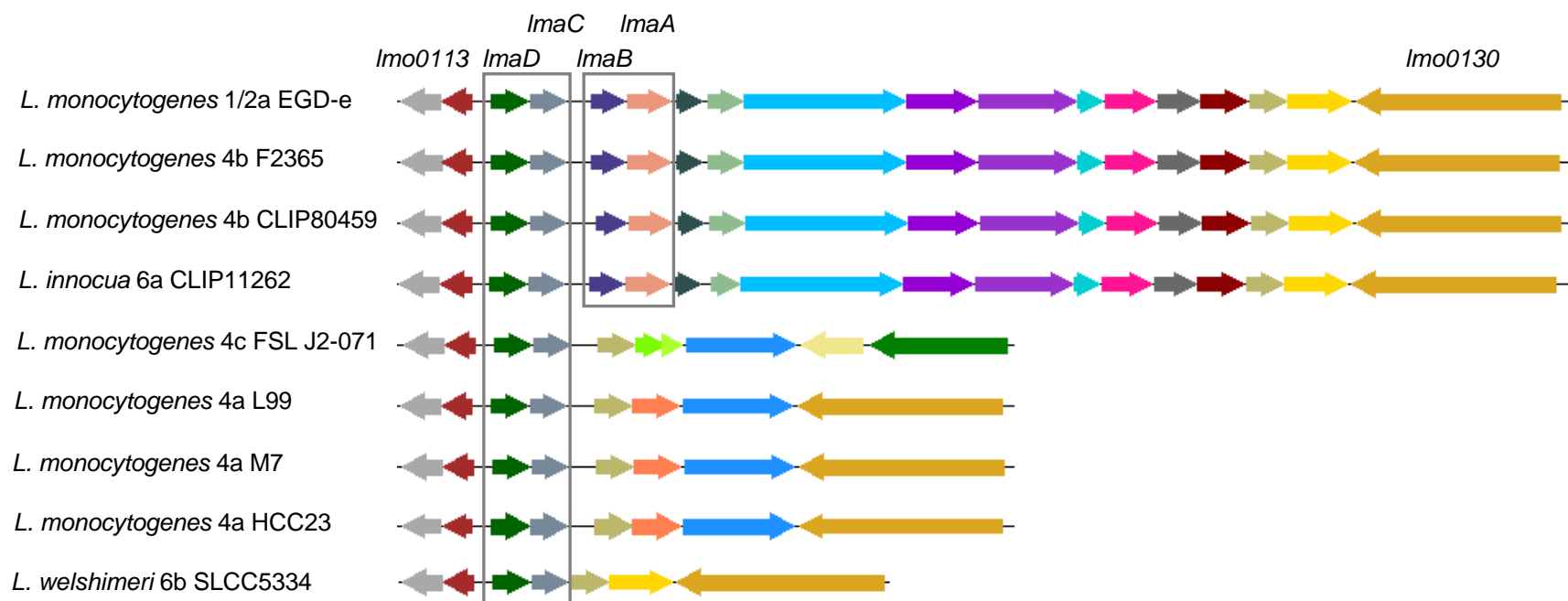

Supplement: Additional file 12 — Figure S2. Comparison of the inlGHE locus in the three listerial lineages. All three genes in this cluster have been absent in the L. monocytogenes 4a L99 genome. [file 1471-2164-13-144-S12.pdf]

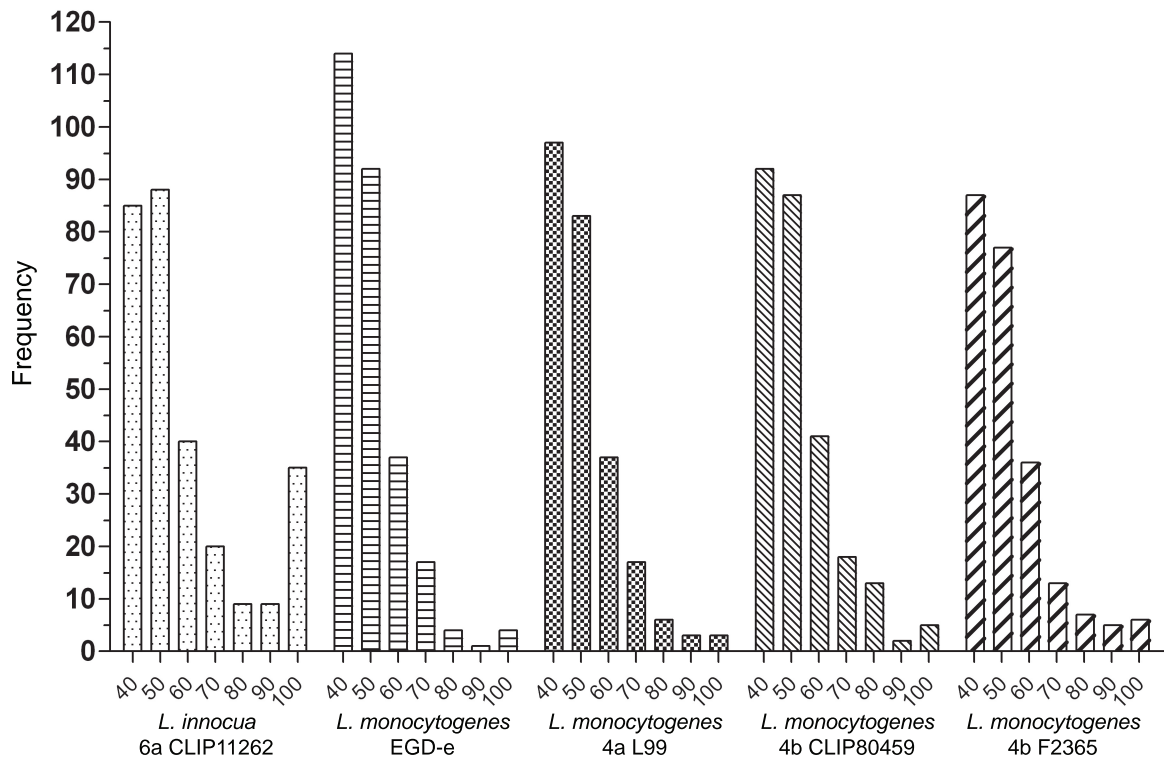

Supplement: Additional file 14 — Figure S4. Frequency of distributions of the percentage identity between all duplicated gene pairs in the Listeria genomes. [file 1471-2164-13-144-S14.pdf]

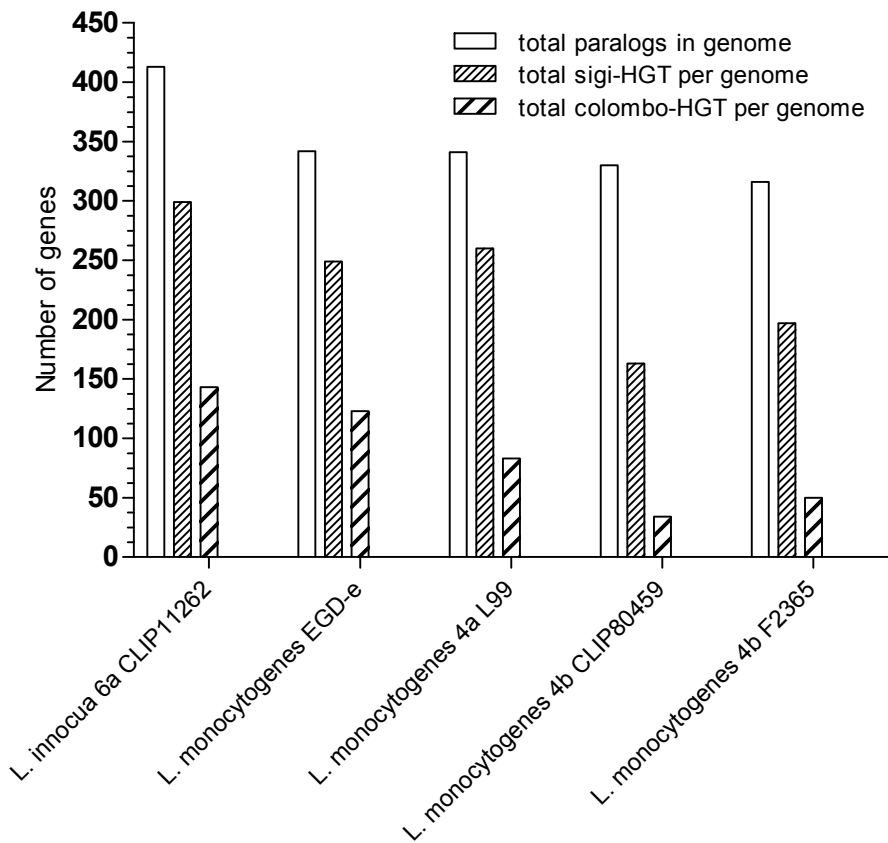

Supplement: Additional file 15 — Figure S5. Gene duplication and horizontal gene transfer in Listeria genomes. [file 1471-2164-13-144-S15.pdf]

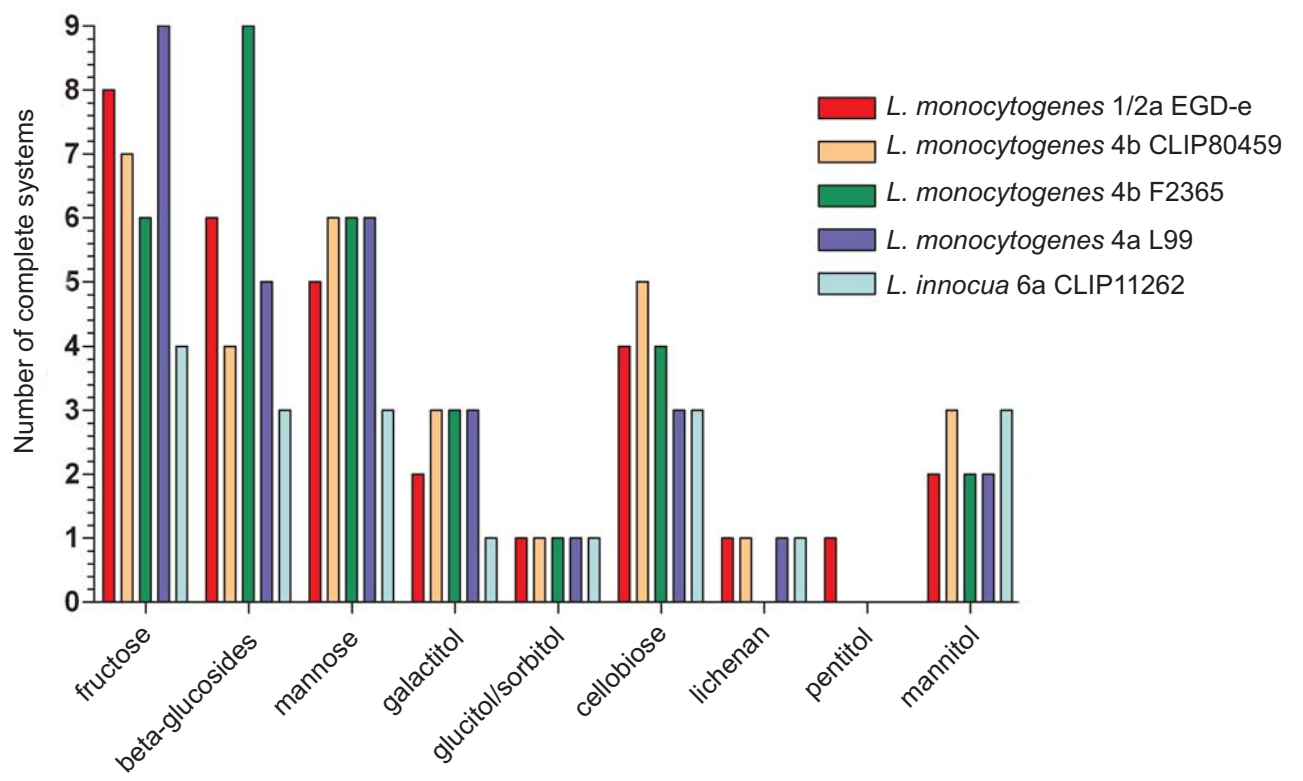

Supplement: Additional file 18 — Figure S8. Partial PTS Systems in L. monocytogenes strains. [file 1471-2164-13-144-S18.pdf]

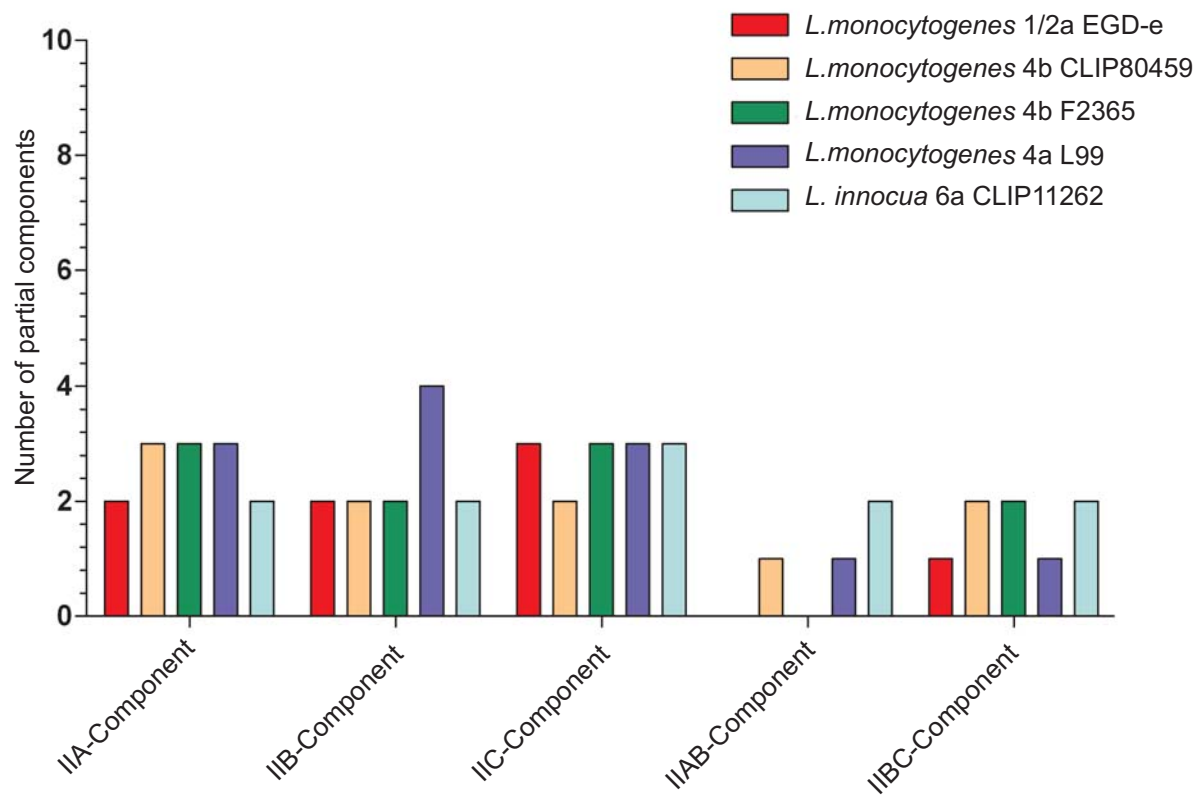

Supplement: Additional file 19 — Text S1. SNP analysis of three listerial lineages. [file 1471-2164-13-144-S19.pdf]

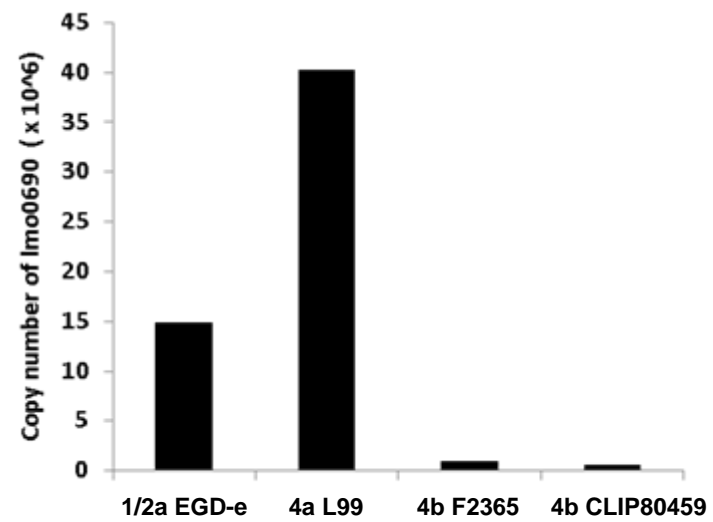

Supplement: Additional file 21 — Text S3. Comparison of two L. monocytogenes 4b strains CLIP80459 and F2365. [file 1471-2164-13-144-S21.pdf]
