# Supplementary material for: Comparative genomics and transcriptomics of lineages I, II, and III strains of Listeria monocytogenes
Source: BMC Genomics. 2012 Apr 24;13:144. doi: 10.1186/1471-2164-13-144 (PMC3464598; doi:10.1186/1471-2164-13-144)
Supplement: Additional file 11 — Figure S1. Comparative analysis of L. monocytogenes ActA protein sequences. [file 1471-2164-13-144-S11.pdf]

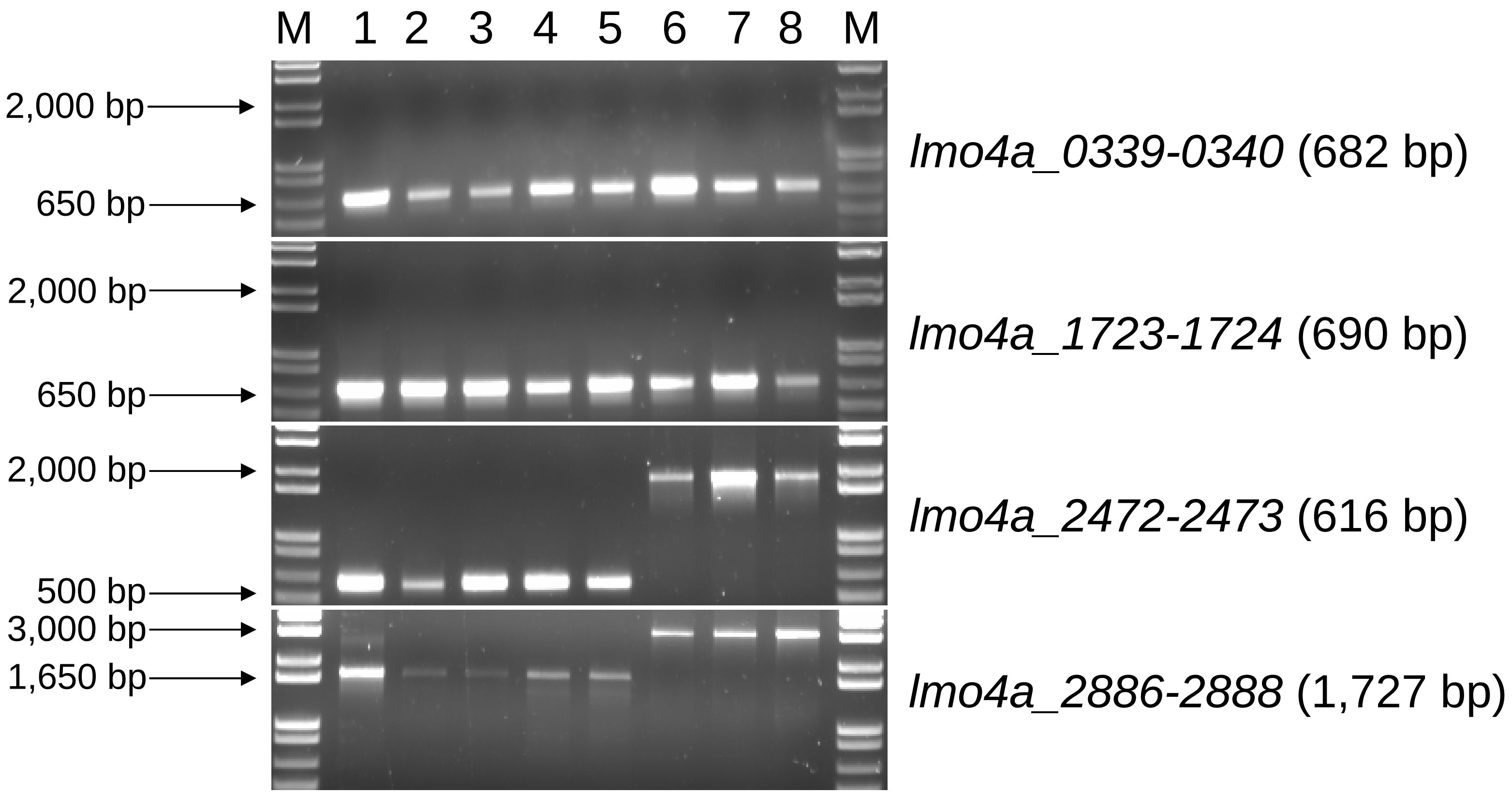

Confirmation of gene regions in *L. monocytogenes* 4a and 4c *strains* compared to those in *L. monocytogenes* 4a L99 using PCR.
Lanes: M, 1 Kb Plus DNA ladder (Invitrogen); 1, *L. monocytogenes* 4a L99; 2, *L. monocytogenes* 4a SLCC2774; 3, *L. monocytogenes* 4a ATCC19114; 4, *L. monocytogenes* 4a SLCC5069; 5, *L. monocytogenes* 4a SLCC5070; 6, *L. monocytogenes* 4c SLCC2376; 7, *L. monocytogenes* 4c SLCC4925 and 8, *L. monocytogenes* 4c SLCC4954. Chromosomal DNA was isolated using DNAeasy blood&tissue DNA isolation kit (Qiagen) and PCR was performed by using the Expand high-fidelity PCR system (Roche) by following the protocol supplied by manufacturer. Primer sequences for all amplicons are the following (in 5'-3' sequence): lmo4a_0399 ACCAAGCGATGGAAGTACGG, lmo4a_0340 CAACAATCCAACCTACCGTA, lmo4a_1723 GATTGATTGCAAGTGGTGTG, lmo4a_1724 CAGCAAGAATGAGACGAGTA, lmo4a_2472 TGCAACTGGTAATTTTGAAGC, lmo4a_2473 CTTATTACACGAGGCGAACA, lmo4a_2886 CCAAGTGATATTTCCGACGT and lmo4a_2888 CGTGTTTGATATTCAGCTGG.
