# Supplementary material for: Comparative genomics and transcriptomics of lineages I, II, and III strains of Listeria monocytogenes
Source: BMC Genomics. 2012 Apr 24;13:144. doi: 10.1186/1471-2164-13-144 (PMC3464598; doi:10.1186/1471-2164-13-144)
Supplement: Additional file 13 — Figure S3. Genome analysis of lmaDCBA region of six listeriae. Comparative analysis was performed using GECO [82] applying bidirectional pairs. [file 1471-2164-13-144-S13.pdf]

Organisms	Array	Start position	Array length	# Spacer	# Repeat	Average spacer length	Distance	Repeat consensus sequence
Listeria monocytogenes 4a HCC23	1	2094578	1781	27	28	35	-	ATTTACATTTCATAATAAGTAGTTAAAAC
	2	2111971	419	6	7	36	15614	ATTTACATTTCACATTAAGTAACTAAAAC
Listeria monocytogenes 4a M7	1	541639	419	6	7	36		GTTTTAGTTACTTAATGTGAAATGTAAAT
	2	557670	1781	27	28	35	15614	GTTTTAACTACTTATTATGAAATGTAAAT
Listeria monocytogenes 4a L99	1	543351	419	6	7	36		GTTTTAGTTACTTAATGTGAAATGTAAAT
	2	559382	1781	28	27	35	15614	GTTTTAACTACTTATTATGAAATGTAAAT
Listeria monocytogenes 4c FSL J2-071	1	519259	670	10	11	35	-	GTTTTAGTTACTTAATGTGAAATGTAAAT
Listeria monocytogenes 1/2a EGD-e	1	544375	289	4	5	36	-	GTTTTAGTTACTTATTGTGAAATGTAAAT
Listeria innocua 6a CLIP11262	1	2768992	696	10	11	30	-	GTTTTGTTAGCATTCAAAATAACATAGCTCTAAAAC
Listeria monocytogenes 4b	-	-	-	-	-	-	-	-
Listeria monocytogenes 4b F2365	-	-	-	-	-	-	-	-
