# Supplementary material for: Comparative genomics and transcriptomics of lineages I, II, and III strains of Listeria monocytogenes
Source: BMC Genomics. 2012 Apr 24;13:144. doi: 10.1186/1471-2164-13-144 (PMC3464598; doi:10.1186/1471-2164-13-144)
Supplement: Additional file 17 — Figure S7. Complete PTS Systems in L. monocytogenes strains. [file 1471-2164-13-144-S17.pdf]

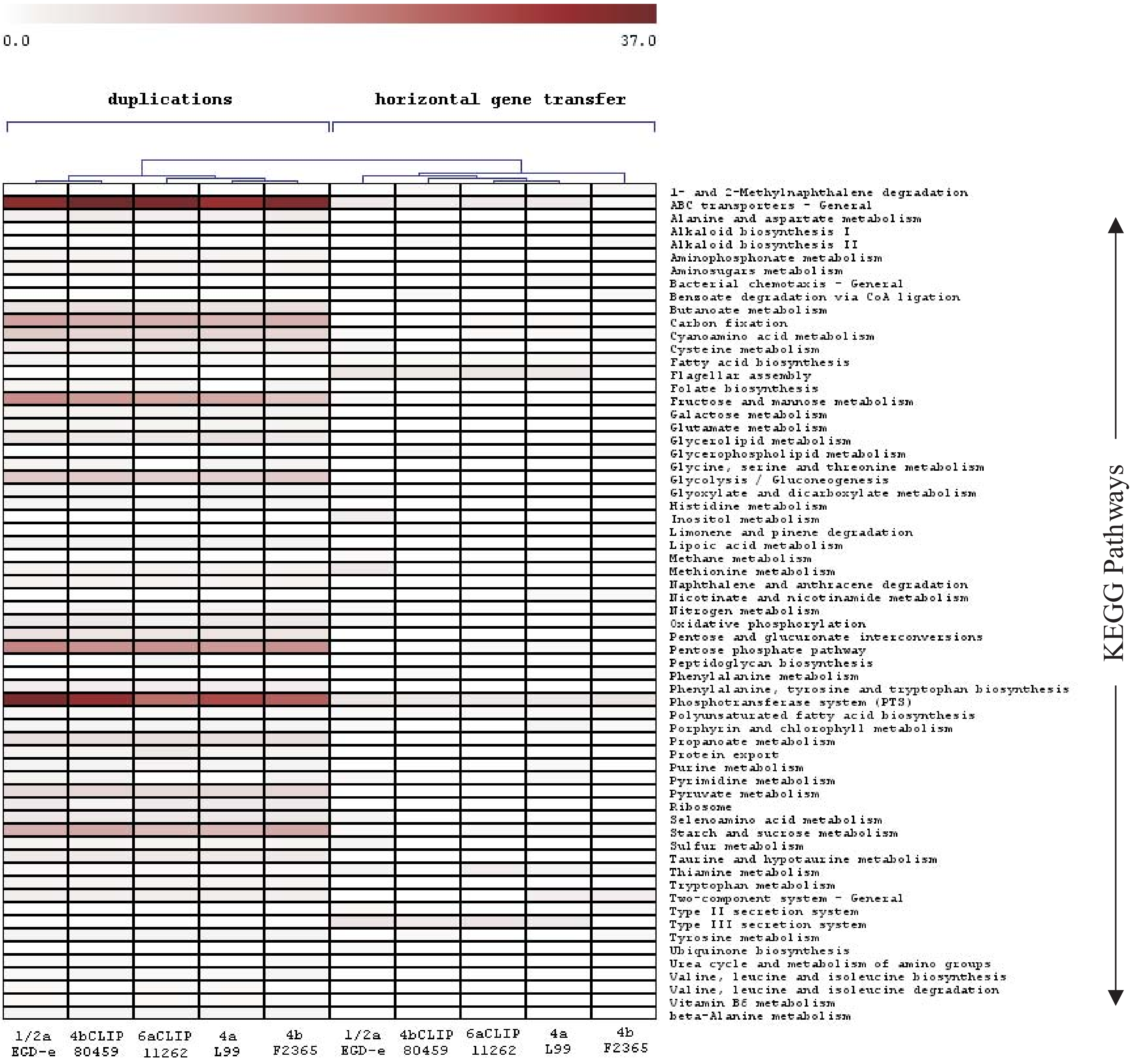

0.0
37.0
duplications
horizontal gene transfer
1- and 2-Methylnaphthalene degradation
ABC transporters - General
Alanine and aspartate metabolism
Alkaloid biosynthesis I
Alkaloid biosynthesis II
Aminophosphonate metabolism
Aminosugars metabolism
Bacterial chemotaxis - General
Benzoate degradation via CoA ligation
Butanoate metabolism
Carbon fixation
Cyanoamino acid metabolism
Cysteine metabolism
Fatty acid biosynthesis
Flagellar assembly
Folate biosynthesis
Fructose and mannose metabolism
Galactose metabolism
Glutamate metabolism
Glycerolipid metabolism
Glycerophospholipid metabolism
Glycine, serine and threonine metabolism
Glycolysis / Gluconeogenesis
Glyoxylate and dicarboxylate metabolism
Histidine metabolism
Inositol metabolism
Limonene and pinene degradation
Lipoic acid metabolism
Methane metabolism
Methionine metabolism
Naphthalene and anthracene degradation
Nicotinate and nicotinamide metabolism
Nitrogen metabolism
Oxidative phosphorylation
Pentose and glucuronate interconversions
Pentose phosphate pathway
Peptidoglycan biosynthesis
Phenylalanine metabolism
Phenylalanine, tyrosine and tryptophan biosynthesis
Phosphotransferase system (PTS)
Polyunsaturated fatty acid biosynthesis
Porphyrin and chlorophyll metabolism
Propanoate metabolism
Protein export
Purine metabolism
Pyrimidine metabolism
Pyruvate metabolism
Ribosome
Selenoamino acid metabolism
Starch and sucrose metabolism
Sulfur metabolism
Taurine and hypotaurine metabolism
Thiamine metabolism
Tryptophan metabolism
Two-component system - General
Type II secretion system
Type III secretion system
Tyrosine metabolism
Ubiquinone biosynthesis
Urea cycle and metabolism of amino groups
Valine, leucine and isoleucine biosynthesis
Valine, leucine and isoleucine degradation
Vitamin B6 metabolism
beta-Alanine metabolism
KEGG Pathways
1/2a EGD-e
4bCLIP 80459
6aCLIP 11262
4a L99
4b F2365
1/2a EGD-e
4bCLIP 80459
6aCLIP 11262
4a L99
4b F2365
