# Supplementary material for: Comparative genomics and transcriptomics of lineages I, II, and III strains of Listeria monocytogenes
Source: BMC Genomics. 2012 Apr 24;13:144. doi: 10.1186/1471-2164-13-144 (PMC3464598; doi:10.1186/1471-2164-13-144)
Supplement: Additional file 20 — Text S2. Differential regulation of glycolysis in L. monocytogenes 4b F2365. [file 1471-2164-13-144-S20.pdf]

Differential regulation of glycolysis in *L. monocytogenes* 4b F2365

This glycolysis operon comprises the following genes (in direction of transcription): the transcriptional regulator *lmo2460* (*cggR*), *lmo2459* (*gap*), *lmo2458* (*pgk*), *lmo2457* (*tpi*), *lmo2456* (*pgm*), and *lmo2455* (*eno*).

From the ten genes of the glycolysis pathway, at least three genes have more than one copy in *L. monocytogenes*, including 3 copies of fructose bisphosphate aldolase, 2 copies of triosephosphate isomerase and 6 copies of phosphoglycerate mutase. While several genes of the glycolytic operon, and several individual genes were downregulated by 1/2a EGD-e, the upregulation of a paralogous triosephosphate isomerase (catalyzing conversion between glyceraldehyde 3-phosphate and dihydroxyacetone phosphate) was observed. In contrast, the 4b F2365 strain showed increased expression of phosphoglycerate mutase. The 4b CLIP80459 strain and the 4a L99 strain also showed upregulation of the phosphoglycerate mutase but they were able to downregulate expression of the other genes in the pathway, in a fashion similar to 1/2a EGD-e. Analysis of the *cggR* binding sequence of the glycolytic operon among the four strains did not reveal any changes (data not shown).
