# Supplementary material for: Comparative genomics and transcriptomics of lineages I, II, and III strains of Listeria monocytogenes
Source: BMC Genomics. 2012 Apr 24;13:144. doi: 10.1186/1471-2164-13-144 (PMC3464598; doi:10.1186/1471-2164-13-144)
Supplement: Additional file 22 — Figure S9. Confirmation of lacking genes encoding surface proteins in four L. monocytogenes 4a strains and three L. monocytogenes 4c strain generated by PCR analysis. [file 1471-2164-13-144-S22.pdf]

Comparison of two *L. monocytogenes* 4b strains CLIP80459 and F2365

Genes involved in cell wall biosynthesis, like *dapF*, *murZ*, *gtcA*, *Lm4b_01101* and *Lm4b_00555* (glycosyl transferases), *Lm4b_01103* (teichoic acid biosynthesis) were also expressed at lower levels in the 4b CLIP80459 strain, indicating reduced cell wall synthesis. Possible reason for the consequent difference in cell invasion may further relate to higher expression of internalin E, but downregulation of internalin A and C by the 4b CLIP80459 strain versus the 4b F2365 strain.

Thioredoxin reductase gene (*trxB*) was found to be upregulated in 4b CLIP80459 strain. This gene is responsible for countering the damaging and potentially cytotoxic effects of oxidative agents. We have also seen that the 4b F2365 strain is possibly unable to reroute its carbohydrate flux efficiently via the pentose phosphate pathway. This observation and that production of *trxB* is increased by 4b CLIP80459 may be another clue leading to the hypothesis that this strain may be more sensitive to oxidative stress than the other *Listeria* strains.

We also observed the higher upregulation of at least three different carbohydrate transporting PTS systems for mannose, beta-glucosides and mannitol in 4b CLIP80459 strain with respect to the 4b F2365 strain. The ability to efficiently utilize host carbohydrates is crucial to intracellular survival. Virulence may be dependent both on number of systems as well as the different types of metabolites that may be processed. Though a number of transport systems (for iron, zinc, and dipeptides) were observed to be upregulated in the 4b F2365 strain, no PTS system was observed to be upregulated compared to the 4b CLIP80459 strain. Some sugar ABC transport proteins were observed, but their specificities are not clear (except for a maltose/maltodextrin ABC transport system with the genes *LMOf2365_0267* and *LMOf2365_0268*). No peptide transporters were upregulated in the 4b CLIP80459 strain compared to the 4b F2365. Both strains upregulated different glycine/betaine transport systems in the host cytosol (*Lm4b_01435* and *LMOf2365_1445*). Importantly, some

components of the propanediol utilization operon (*Lm4b_01159* and *Lm4b_01169*), which has been shown to be crucial for intracellular survival of *Salmonella*, were expressed at higher levels in the 4b CLIP80459 strain than in the 4b F2365.

Among virulence genes, *mpl* and *clpE* were expressed at higher levels by 4b CLIP80459 while internalin A and internalin C were expressed at lower levels.

In surface proteins, several lipoproteins were upregulated by 4b CLIP80459 (*Lm4b_00178*, *Lm4b_00331*, *Lm4b_01659*, *Lm4b_02246* and *Lm4b_02385*), one protein containing GW modules (*Lm4b_01220*) and one containing a LysM domain (*Lm4b_02353*). The strain 4b F2365 also upregulated several lipoproteins (*LMOf2365_0267*, *LMOf2365_0557*, *LMOf2365_0605*, *LMOf2365_1989*, *LMOf2365_2615* and *LMOf2365_2830*), some LPXTG motif containing proteins (*LMOf2365_0148* and *LMOf2365_0605*) and a LysM domain containing protein (*LMOf2365_1321*). In this manner, there are possibly considerable differences in the surface proteins presented to the host cytosol even by these closely related strains.
