# Supplementary material for: Comparative genomics and transcriptomics of lineages I, II, and III strains of Listeria monocytogenes
Source: BMC Genomics. 2012 Apr 24;13:144. doi: 10.1186/1471-2164-13-144 (PMC3464598; doi:10.1186/1471-2164-13-144)
Supplement: Additional file 23 — Figure S10. Intracellular flagellin expression data of L. monocytogenes 1/2a EGD-e, L. monocytogenes 4a L99, L. monocytogenes 4b CLIP80459 and L. monocytogenes 4b F2365 generated by qRT-PCR analysis. [file 1471-2164-13-144-S23.pdf]

| Function | Name | Locustag | Lmo number | Annotation |
|---|---|---|---|---|
| metabolosome | *eutS* | STM2470 | *lmo1144* | propanediol utilization protein PduU |
| unknown | *eutP* | STM2469 | *lmo1145* | propanediol utilization protein PduV |
| unknown | *eutQ* | STM2468 | *lmo1187* | unknown |
| corrinoid | *eutT* | STM2467 | *lmo1181* | ethanolamine utilization cobalamin adenosyltransferase |
| adenosyltransferase | *eutD* | STM2466 | *lmo2103* | Pta protein (EC 2.3.1.8) |
| phosphotransacetylase | *eutM* | STM2465 | *lmo1180* | microcompartments protein |
| metabolosome | *eutN* | STM2464 | *lmo1163* | carboxysome structural protein |
| adldehyde dehydrogenase | *eutE* | STM2463 | *lmo1165* | CoA-dependent propionaldehyde dehydrogenase |
| chaperone | *eutJ* | STM2462 | *lmo1161* | putative heatshock protein |
| alcohol dehydrogenase | *eutG* | STM2461 | *lmo1166, lmo1171* | propanol dehydrogenase |
| permease | *eutH* | STM2460 | *lmo1186* | permease, transport protein |
| EAL reactivase | *eutA* | STM2459 | *lmo1174* | EAL reactivase |
| EA ammonia-lyase | *eutB* | STM2458 | *lmo1175* | EAL large subunit |
| EA ammonia-lyase | *eutC* | STM2457 | *lmo1176* | EAL small subunit |
| metabolosome | *eutL* | STM2456 | *lmo1177* | microcompartments protein |
| metabolosome | *eutK* | STM2455 | *lmo1159* | microcompartments protein |
| transcription activator | *eutR* | STM2454 | *lmo1189* | HTH AraC family transcriptional |
