# Supplementary material for: Comparative genomics and transcriptomics of lineages I, II, and III strains of Listeria monocytogenes
Source: BMC Genomics. 2012 Apr 24;13:144. doi: 10.1186/1471-2164-13-144 (PMC3464598; doi:10.1186/1471-2164-13-144)
Supplement: Additional file 24 — Figure S11. List of gene duplication in Listeria genomes. [file 1471-2164-13-144-S24.pdf]

Primer	Sequence 5' -> 3'
lmaB-P1	CTGGAGCATTGTTCTCACTGCTTCC
lmaB-P2	GGCGCTTTACCTGCTTCGAC
lmaB-P3	TTTGAAGTAAGTATTTGTCG
lmaB-P4	TTCTGCTGGTGCTACAGGTG
lmaD-P1	GCAATGATGTCATCAAGCGG
lmaD-P2	TAAAGCTTTCTATCCATCCATTCCC
lmaD-P3	GGCGCTTTACCTGCTTCGAC
lmaD-P4	CTGGAGCATTGTTCTCACTGCTTCC
